# Atomistic Picture for the Folding Pathway of a Hybrid-1 Type Human Telomeric DNA G-quadruplex

**DOI:** 10.1371/journal.pcbi.1003562

**Published:** 2014-04-10

**Authors:** Yunqiang Bian, Cheng Tan, Jun Wang, Yuebiao Sheng, Jian Zhang, Wei Wang

**Affiliations:** 1 National Laboratory of Solid State Microstructure and Department of Physics, Nanjing University, Nanjing, China; 2 High Performance Computing Center, Nanjing University, Nanjing, China; University of Maryland, Baltimore, United States of America

## Abstract

In this work we studied the folding process of the hybrid-1 type human telomeric DNA G-quadruplex with solvent and 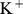 ions explicitly modeled. Enabled by the powerful bias-exchange metadynamics and large-scale conventional molecular dynamic simulations, the free energy landscape of this G-DNA was obtained for the first time and four folding intermediates were identified, including a triplex and a basically formed quadruplex. The simulations also provided atomistic pictures for the structures and cation binding patterns of the intermediates. The results showed that the structure formation and cation binding are cooperative and mutually supporting each other. The *syn/anti* reorientation dynamics of the intermediates was also investigated. It was found that the nucleotides usually take correct *syn/anti* configurations when they form native and stable hydrogen bonds with the others, while fluctuating between two configurations when they do not. Misfolded intermediates with wrong *syn/anti* configurations were observed in the early intermediates but not in the later ones. Based on the simulations, we also discussed the roles of the non-native interactions. Besides, the formation process of the parallel conformation in the first two G-repeats and the associated reversal loop were studied. Based on the above results, we proposed a folding pathway for the hybrid-1 type G-quadruplex with atomistic details, which is new and more complete compared with previous ones. The knowledge gained for this type of G-DNA may provide a general insight for the folding of the other G-quadruplexes.

## Author Summary

G-quadruplexes are high-order DNA/RNA structures. They are involved in the regulation of telomere maintenance, DNA replication, transcription and translation, and are also attractive drug designing targets for treating cancers and promising building blocks for molecular nanodevices. The knowledge of their formation process will improve our understanding of how they achieve their functional structures and then facilitate designing of artificial G-quadruplexes with novel functions. The study of their formation process is also of academic importance, since they involve many different physical chemical factors or interactions, including the hydrogen bonds, the electrostatic effect associated with metal ions, and the *syn/anti* reorientation of the glycosidic bonds. These make the G-quadruplex a fascinating model system for studying the structure formation of bio-molecules. Furthermore, the study of their formations may enrich the free energy landscape theory that has been well developed for protein folding, but yet to be verified in the other biomolecular systems. Here we computationally study the folding process of the hybrid-1 type human telomeric DNA G-quadruplex and infer a new folding picture, which may also cast a light to the formation of the other G-quadruplexes.

## Introduction

G-quadruplexes are high-order DNA or RNA structures formed from guanine-rich sequences, and their building blocks are G-tetrads that arise from Hoogsten hydrogen-bonding between four guanines. The G-tetrads stack on top of each other and form four-stranded helical structures. Bioinformatics analysis suggests that G-quadruplex motifs are prevalent in genomes. Recently, experimental evidence is accumulating for the in vivo presence of G-quadruplexes in DNA telomeres, in gene promoter regions [1], and even in messenger RNAs [2], [3], suggesting that they are involved in the regulation of telomere maintenance, replication, transcription and translation. G-quadruplexes are also attractive drug designing targets for treating cancers and platforms for delivering drugs [4]. Despite of their functional importance, the folding processes by which they achieve the functional structures have not been well understood as that of DNA and RNA duplexes [5]–[12]. It is believed that there are significant differences between G-quadruplexes and duplexes in the balance of forces, mainly the hydrogen bonds and electrostatic interactions [13]. Therefore, the study of the folding of G-quadruplex will improve our understanding of the balance between different forces in determining the structures and dynamics of such a typical folded oligonucleotide, and may facilitating designing new quadruplexes with novel functions. Moreover, the knowledge may enrich the energy landscape theory that has been well developed for protein folding, but yet to be verified in the other biomolecular systems. However, the folding of G-quadruplexes is a difficult problem due to its sensitivity to the terminal nucleotides, the dependence on ion types and concentration, and particularly due to the little known interplay between metal ions and folding dynamics; the *syn/anti* reorientations of the glycosidic bonds of the nucleotides further complicate the folding process.

There are lots of experimental works on different forms of G-quadruplexes, studying their native structures, thermodynamical properties, folding kinetics and cooperativity, as well as the roles of ions in the stability and folding process. A detailed discussion of these works is beyond the scope of this article and can be found in several excellent reviews [13]–[19]. Recently, new progress has been made on the folding intermediates of DNA quadruplexes [20]–[23], particularly those achieved by single-molecular techniques including optic tweezers and magnetic tweezers [24]–[26]. For example, Wei *et al.* investigated the folding kinetics of human telomeric G-quadruplexes using magnetic tweezers and detected a G-triplex [25]; they also observed reversible transitions from the G-quadruplex to the G-triplex as well as from the G-triplex to the unfolded coil, and then suggested that the G-triplex is an in-pathway intermediate. Molecular modeling and simulations are able to complement experiments by providing much detailed information or insights [27]–[31]. For example, Sugiyama *et al.* systematically investigated the intermediates of human telomeric G-quadruplexes using *ab initio* calculations and MD simulations; the folding pathways and the roles played by 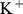 ions were discussed [29], [30]. Limongelli *et al.* studied the folding of a 15-mer G-quadruplex using metadynamics; they identified a stable G-triplex and then validated it with a number of experiments [32]. Despite of many pioneer works, the atomistic picture for the folding pathways of quadruplexes is still lacking due to the temporal and spatial resolution limits of experimental techniques, the exclusion of conformation dynamics or entropies in theoretical analysis, or insufficient sampling of the phase space in previous all-atom computer simulations.

In this work we studied the folding process of a 24-nt human telomeric DNA sequence 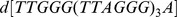 (PDB ID 2GKU) (Figure S1) [33] with explicitly modeled solvents and ions using an advanced sampling technique and large-scale simulations. This sequence was selected since it forms a unique native structure of hybrid-1 type in KCl solution at room temperature and has many experimental results to be compared with [23], [30], [33], [34]. The folding time of this sequence was measured to be longer than 10 ms by stopped-flow and spectroscopic techniques [34], well beyond the timescale of traditional all-atom MD simulations. To overcome the barrier crossing problem, we combined the power of large-scale simulations and a novel advanced sampling technique named bias-exchange metadynamics, which is very efficient at accelerating barrier-crossing events by periodically modifying the effective energy felt by the system with small repulsive Gaussian potentials and thus enforcing the escape from local minima [35]. For a further acceleration of the sampling and increase of its coverage in the phase space, multiple (six) copies of metadynamics were run simultaneously with each biased on a different collective variable (CV) [36]. The conformations and velocities of different replicas were allowed to exchange periodically according to a metropolis criterion. From the data obtained by bias-exchange metadynamics, we calculated the free energy landscape, identified several intermediate states, and further studied their stabilities and dynamics by performing massive conventional MD simulations. Based on the above results we proposed an atomistic picture for the folding process of the hybrid-1 type G-DNA and discussed its relevance to the previous experimental and theoretical results.

## Results

### The free energy landscape and the intermediates

The convergence of the bias-exchange metadynamics was tested by monitoring the random walk of the replicas in their CV spaces, the exchange probability as a function of simulation time, and the evolution of FEL during simulation (Figure S3). For the four biased replicas, the CVs sampled all the possible values of Q and 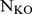, and a broad region of dRMSD (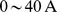) and 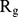 (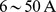); and the replica walked back and forth many times in the relevant space. These features indicated that the simulation sampled a sufficient large region of CV spaces. The number of successfully exchanged events was almost linear as a function of time for all replicas, showing that the exchange happened at a steady rate throughout the simulation. The average exchange probabilities were in the range of 4–5% for the four biased replicas and about 21% for the neutral replicas. The lower values for four biased replicas were expected since they were biased at different CVs and had very different energetics. The FELs were calculated solely from the neutral replicas to avoid potential problems from the applied biases in the other replicas. It was found that the general shape of the FELs did not change after 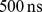, and the two FELs calculated respectively from two neutral replicas at 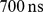 were almost indistinguishable. Besides, the highest free energy barrier between basins was around several kcal/mol, reflecting a good sampling quality of the relevant phase space. The FELs at 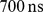 will be used for the following analysis.

**Figure 1:**
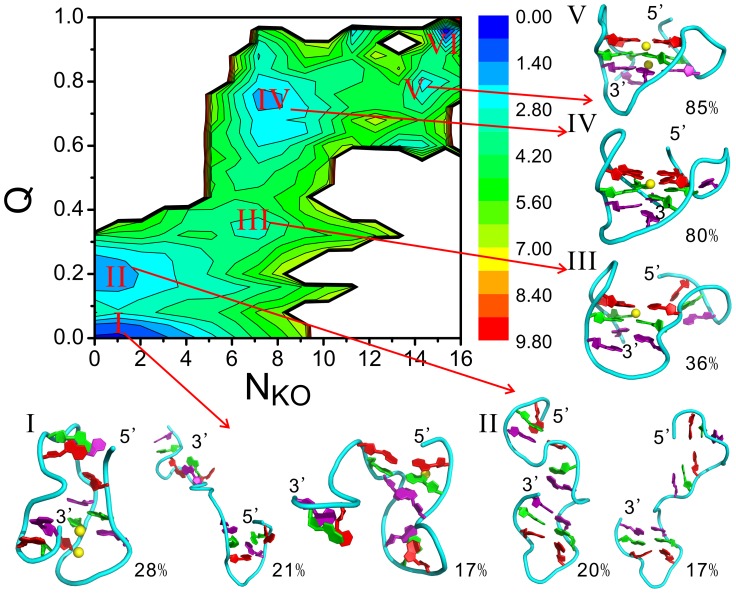
The free energy landscape and the representative structures of the basins of attraction. The unit of the free energy is kcal/mol. Multiple representative structures are given for the first two basins, due to the their heterogeneous nature. The populations of the representative structures within their respectively basins are roughly estimated and indicated by the numbers beside them. The three G-tetrads in the structures are colored red, green, and purple, respectively. This color code will be used throughout the whole text unless otherwise indicated. The 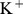 ions bound to DNA are shown as yellow spheres.

The free energy landscape shown in Figure 1 roughly manifests a diagonal shape, indicating the cooperativity between the formation of native contacts and the binding of metal ions. From the FEL six basins of attraction are identified and labeled from I to VI, respectively. Their representative structures are also shown in the figure, obtained based on a clustering analysis [37] of the belonging conformations, which are determined using their CVs. For the first basin it is found that the structures are pretty heterogeneous. For example, the largest cluster has a rather compact structure, i.e., the first two G-repeats (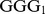 and 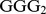) roughly form a hairpin, upon which docks the 3′ terminal via non-native interactions. The second and third largest clusters are both characterized by hairpins, however, formed between 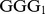 and 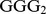 and between 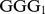 and 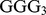, respectively. Most stable interactions observed in the first basin are non-native ones, supported by the hydrogen bond map averaged on all the structures belonging to this basin (Figure S4). The 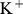 ions binding on the G-DNA are weak, with the binding probabilities generally lower than 0.15. Besides, the binding probabilities are almost uniform on all nucleotides; there is no specific binding detected (Figure S4). Based on the above analysis, the basin-I is designated as the denatured state.

The last basin (basin-VI) occupies a narrow area and is characterized by the highest values of Q and 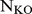 among all basins. Plus, clustering analysis showed that the belonging structures are homogenous and similar to the native one. Therefore the basin-VI is concluded to be the native state of the G-DNA.

In addition to the basin-I and basin-VI, there are other four basins of attraction on the FEL. Obviously these are intermediate states and hold the key for understanding the folding process of the G-DNA. For a better characterization of these intermediates, we feel that a clustering analysis of the BEMD data is not accurate enough, since it is not trivial to determine the width of a basin and whether a structure belongs to that basin solely based on the CVs, due to possible overlaps between basins in a low-dimensional projection of the free energy landscape. Therefore we further performed multiple conventional MD simulations initialized from these intermediates. Such simulations are free of the above mentioned problems, and most importantly, they are able to provide true dynamics of the intermediates, which is lost in BEMD due to the added potentials. In the following sections we will discuss the structures and dynamics of the intermediates by combining the data from BEMD with that from conventional simulations.

### The structure and dynamics of the intermediates

**Figure 2:**
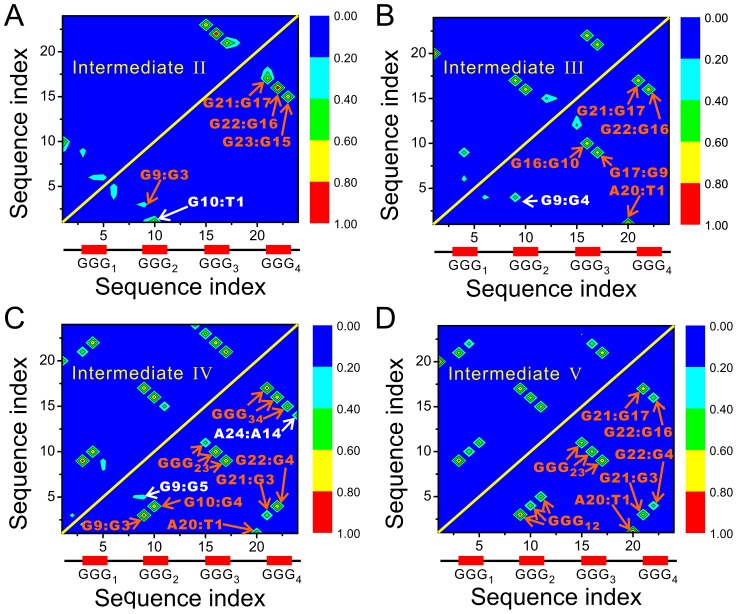
The hydrogen bond maps for the intermediates. (A)–(D) are for the intermediate-II, III, IV, and V, respectively. The formation probabilities shown here are averaged on all the structures collected from multiple conventional MD simulations. Their values are indicated by the color scales. The hydrogen bonds pointed by the red arrows are native ones that exist in the native structure, while those pointed by the white arrows are non-native ones.

**Figure 3:**
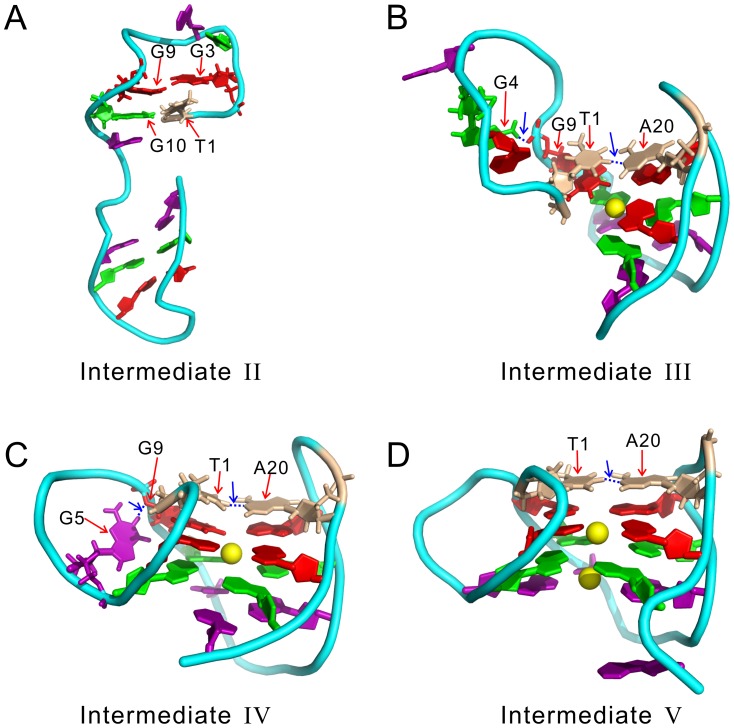
The detailed tertiary structures of the intermediates. (A)–(D) are for the intermediate-II, III, IV, and V, respectively. The structures are taken from the largest cluster in the corresponding conventional MD trajectories. They are slightly different from that shown in Figure 1, which are obtained from BEMD simulations. The non-native hydrogen bonds are plotted as blue dashed lines and pointed by blue arrows. The 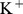 ions bound to DNA are shown as yellow spheres.

**Figure 4:**
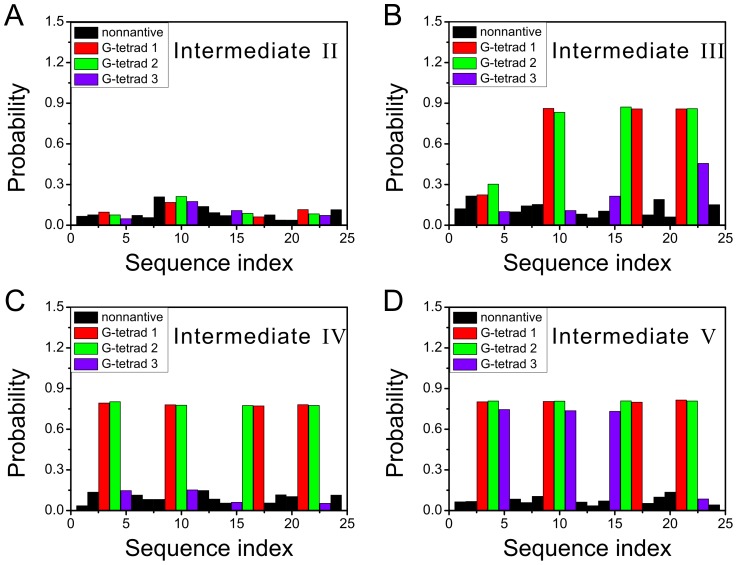
The 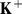 ion binding probabilities for the four intermediates. (A)–(D) are for the intermediate-II, III, IV, and V, respectively. The color code is the same as in Figure 1, i.e., the red, green, and purple histograms correspond to the three G-tetrads, respectively. The black histograms indicate the binding probabilities on the non-native sites.

The structure of the intermediate-II is heterogeneous, mainly characterized by a well formed hairpin at 3′-terminal and an unstable hairpin at the 5′-terminal according to Figure 1. Conventional MD simulations initialized from the largest cluster confirmed such an observation. As shown by the hydrogen bond map in Figure 2(A) and the detailed structure in Figure 3(A), the intermediate-II is compose of a well-formed native hairpin between the G-repeats 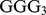 and 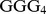 (shorted as 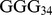 hereafter) and a non-native hairpin formed by the first two G-repeats via G9∶G3 and G10∶T1; and the interactions between two hairpins are ignorable. Dynamically, the structures are under constant fluctuations, with the RMSDs up to 1 nm with respect to their initial conformations. The fluctuations are mainly associated with relative motions between two hairpins (Figure S5 and Video S1). The consistence of the conventional simulations with the BEMD data suggests that the former has covered the most relevant phase space of the intermediate-II, although the initial structures were chosen only from the largest cluster, whose population was about 20% in this intermediate. Besides, it is interesting to note that 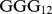 is in an antiparallel conformation, in contrast to its parallel conformation in the native structure. The latter structure is probably not stable in this stage without the supporting from the nearby interactions, due to the tension associated with the parallel conformation and the reversal loop. The ion binding pattern of this structure shown in Figure 4(A) is similar to that of the denatured structures, i.e., the binding probabilities are low and almost distributed evenly on all nucleotides. There is no strongly binding sites observed.

The intermediate-III is a native triplex composed of the last three G-repeats (denoted as 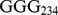 hereafter), revealed by BEMD (Figure 1) and confirmed by multiple conventional MD simulations. As shown in Figure 2(B), there are two large groups of hydrogen bonds, including that between the G-repeat 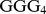 and 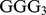 (G21∶G17 and G22∶G16), and between 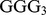 and 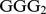 (G17∶G9 and G16∶G10). The detailed structure in Figure 3(B) consistently shows that 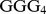 is spatially close to 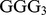, and 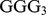 is close to 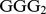. The initial structures for running conventional MD simulations represent roughly 36% populations in the intermediate-III, however, the trajectories still cover a broad region of phase space (Figure S9). Conventional simulations demonstrate two different dynamics that drive the G-DNA toward different destinations. The first kind is a docking of the 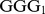 on the triplex, which essentially makes the structure transform into the intermediate-IV (Figure S9 and Video S2). Another dynamics is characterized by a flanking motion of the 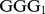 with respect to the triplex, constrained by a native base pair A20∶T1 and an non-native interaction G9∶G4 (Figure 2–3 and Video S3). These two interaction pull the first G-repeat close to the triplex so that it will not drift away from the triplex. The flanking motion keeps the G-DNA in the original basin and results in a fluctuating structure, which will eventually transform into the intermediate-IV via the first kind of dynamics described above.

In the intermediate-III, metal ion binding pattern becomes interesting. As shown in Figure 3 and 4, a 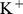 ion is trapped between the first and second G-tetrads, resulting in high ion binding probabilities of almost 90% of the nearby nucleotides. Clearly, the binding of a positive ion compromises the strong negative charges along the backbone and further stabilizes the base pairs by coordinating the O6 atoms of the nearby bases. The second 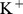 ion between the second and third G-tetrad seen in the native structure is absent in the intermediate-III, therefore the nearby native base pairs G23∶G15 and G15∶G11 are hardly detectable, although the three nucleotides are almost in position (Figure 2(B) and Figure 3(B)).

The structure of the intermediate-IV is characterized by a incomplete docking of 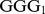 on the triplex 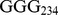, supported by both BEMD and conventional simulations (Figure 1–3). The last nucleotide G5 in 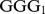 does not reach its correct position in the third G-tetrad but forms non-native hydrogen bonds with G9 instead. The G3 and G4 nucleotides in 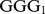, however, bind correctly to the trapped 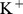 ion in the central channel and their ion binding probabilities increase to about 80% from below 30% (Figure 4), leading to a basically formed quadruplex. At this folding stage, the lower ion binding site in the central channel is still unoccupied, although seven out of eight of its nearby nucleotides are in position. Dynamically speaking, the whole structure is very stable, indicated by the not-larger-than 0.25 nm RMSDs with respect to the initial structures (Figure S7). This dynamics is believed to be representative of that of the intermediate-IV since the initial structures for running conventional simulations represent 80% population of the basin.

The intermediate-V is different from the preceding intermediates primarily in the trapped 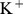 ion in lower site in the quadruplex channel (Figure 1). The trapped ion results in a further strengthening of the native base pairs and increase of ion binding probabilities of the nucleotides in the third G-tetrad (Figure 2–4). The structure is very similar to the native one and thus the intermediate should be viewed as a sub-state of the native basin of attraction. Indeed, we observed two direct folding trajectories from this intermediate to the native states in the conventional simulations (Figure S8 and S9).

### The *syn/anti* reorientations of the glycosidic bonds

**Figure 5:**
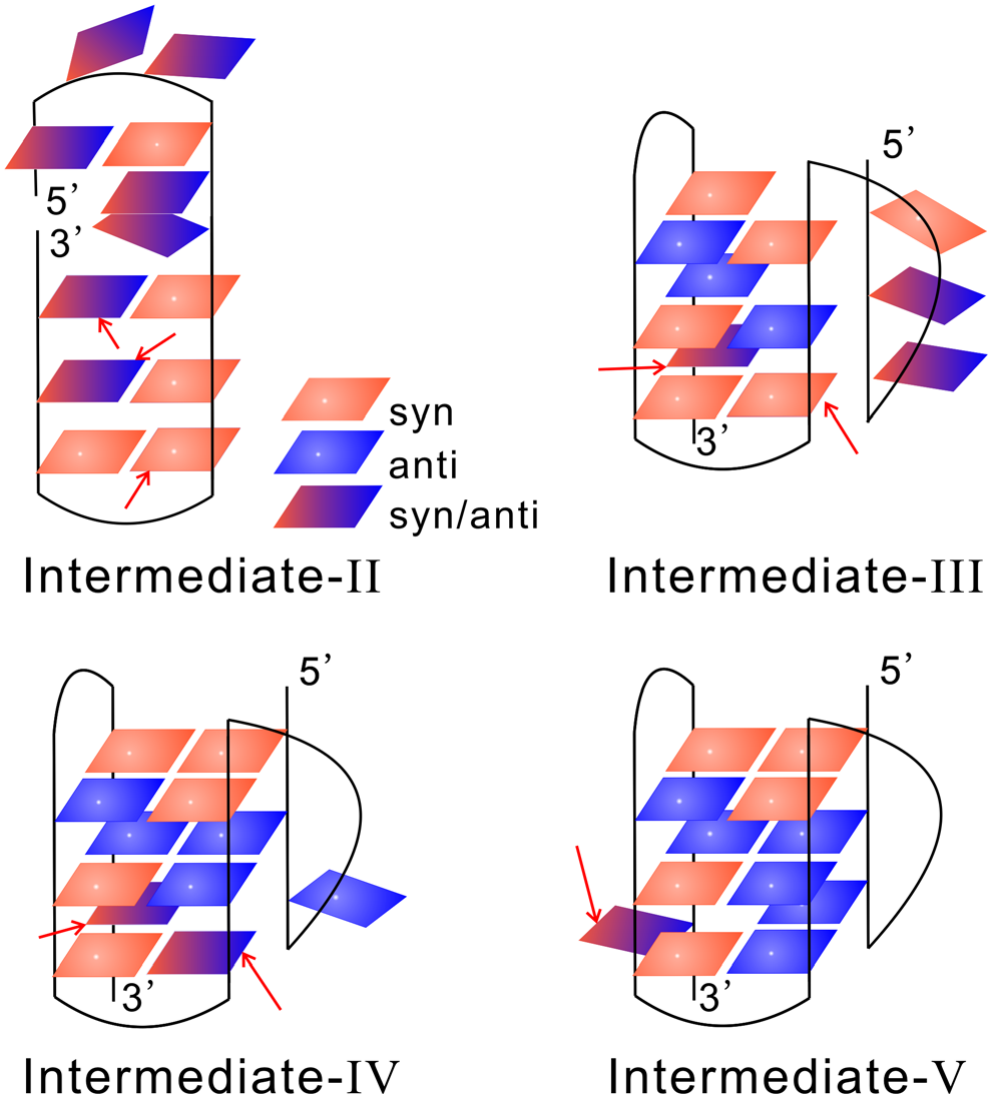
The *syn/anti* patterns of the intermediates. The bases that have formed native hydrogen bonds in between are plotted side-by-side and in the same plane. The red squares denote the nucleotides with 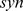 configurations, the blue the 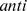; and the gradient color indicates a fluctuating configuration between 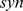 and 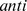. The nucleotides indicated by arrows correspond to either fluctuating (with gradient color) or wrong *syn/anti* configurations. Here by wrong we mean that they retain a *syn/anti* configuration different from the native one. The details of the trajectories are given in Figure S11, S12, S13, S14.

The folding of G-DNA is complex partially due to the involvement of the *syn/anti* reorientations of the glycosidic bonds. To reveal how such motions interplay with the folding process, we analyzed the *syn/anti* patterns and dynamics of the intermediates based on multiple conventional MD simulations. The torsion angle used to determine the *syn/anti* configurations for a specific glycosidic bond was calculated based on the following four atoms: O4′ and C1′ in the sugar ring, and N9 and C8 in the base. The results are shown in Figure 5 and in Figure S11, S12, S13, S14. It can be seen that in four intermediates the glycosidic bonds generally take correct *syn/anti* configurations when the corresponding nucleotides form native and stable base pairs with the others. Here by correct we mean the glycosidic bonds take the same configurations as in the native structure. However, fluctuating glycosidic bonds are also observed. For example, in the intermediate-II there are two nucleotides (G22 and G23) fluctuating between 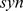 and 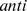 configurations although they are within the basically formed 3′-terminal hairpin. Even after the structure transforms to the intermediate-III, G23 is still under fluctuation. The typical time scale for the *syn/anti* transitions is of order of ten nanoseconds, according to conventional MD simulations (Figure S11, S12, S13, S14). Interestingly, two nucleotides with wrong *syn/anti* configurations are observed although they have formed base pairs with others, which are G17 in the intermediate-II and G11 in the intermediate-III, tentatively attributed to their outer position in the formed structure and associated larger flexibility. From the intermediates-III to V, more and more stable base pairs are formed and the fluctuating bonds become fewer accordingly. In the last two intermediates, we also observed fluctuating bonds but no wrong *syn/anti* configurations.

## Discussion

The combined power of bias-exchange metadynamics and large scale conventional MD simulations enabled us to explore the free energy landscape of the DNA G-quadruplex and the structure and dynamics of the intermediates. The relevance of the results described above to the previous experimental and theoretical data is discussed in the following sections.

Recently, the existence of a triplex as a folding intermediate in several different quadruplexes has been established by many experimental approaches, including CD, DSC, and ITC analysis [20], FRET [23], optic tweezers [24], and magnetic tweezers [25]. However, the detailed structure of the triplex, particularly the binding patterns of the associated metal ions, is still unclear due to temporal and spatial resolution limits of experimental techniques. The triplex detected in our simulations (the intermediate-III) is relevant to that detected in previous experiments. For example, our triplex is characterized by a docking of 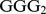 on the hairpin 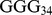, with the first G-repeat at the 5′ terminal fluctuating around. This structural feature has also been observed in the thermal denaturation experiments of several human telomere DNA sequences including Tel22 and 2GKU by Gray *et al.* [23], who found that these two DNAs have common unfolding pathways and the intermediate triplex states have greater solvent exposure of the 5′-segment. The folding/unfolding of Tel22 in the presence of 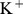 ions has also been studied by another group using DSC and CD measurements; they confirmed the existence of a triplex as intermediate state and determined a release of 1.5 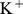 ions from the folded to the triplex states [20]. As a comparison, our calculation shows that the average numbers of bound 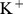 ions in the triplex and in the native states are 1.2 and 3.0, respectively (Figure S10); the difference of 1.8 ions agrees quite well with the experimental value. Furthermore, Mashimo *et al.* systematically calculated the energies of various possible topologies of triplex using *ab initio* molecular dynamics and fragment molecular orbital method [30], and then for the type-1 quadruplex such as 2GKU they suggested a triplex that has a similar structure to ours. Therefore the triplex detected in our computations is relevant to previous experimental and theoretical ones. Moreover, our analysis provides more atomistic details on its structure, particularly in the patterns of metal ion binding.

In the folding studies of the hybrid-1 type G-DNAs, the formation time and folding dynamics of the parallel conformation in the 5′-terminal and the associated reversal loop are always a myth. In previous literatures, it was often suggested that these local structures form at the end of the folding stage via a flip of the first G-repeat. However, there is little direct proof supporting this suggestion. Here thanks to the powerful BEMD, we observed two intermediates that provided insights into the underlying dynamics. In the structure of the intermediate-III, the first G-repeat 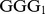 is constrained by the interaction A20∶T1 and G9∶G4 in such a place, that only a flanking motion of 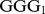 with A20∶T1 as a pivot is needed to form the parallel conformation (Figure 3). In the conventional MD simulations started from this intermediate, we indeed observed two direct trajectories that transformed from the intermediate-III to IV with such a motion (Figure S6 and Video S2). As a result, in the intermediate-IV the parallel conformation and the reversal loop have been mostly formed (Figure 3). Therefore the formation of the local structures is not a once-for-all event occurring in the final folding stage, as often implicated in previous literatures. Instead, the formation starts early with the triplex (the intermediate-III), and is basically finished when the triplex transforms into the quadruplex (the intermediate-IV); and the final formation is accomplished after the trapping of the second 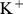 ion in the central channel (the intermediate-V). We believe that the new picture can be easily verified by experiments, since it suggests that the A20∶T1 and G9∶G4 interactions play a key role during the transition from the triplex to the quadruplex. It is highly possible that a knockout of these interactions will significantly impede the formation of the reversal loop and slow down the folding rate of the G-DNA.

Early folding events are also important for understanding the whole folding process [38], [39]. Previously, Mashimo and colleagues proposed that the type-1 quadruplex first folds into the hairpin 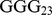 and then to the triplex 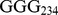 based on *ab initio* calculations and molecular simulations [30]. Although our work agrees with theirs on the formation of 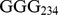 as an intermediate, it suggests a different initial structure, 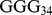 versus 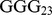. To determine which structure is more kinetically connected to the triplex, we performed 10 high temperature unfolding simulations starting from the native structure (Figure S15). It was found that 8 of them unfold into structures containing 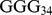, while only one into that containing 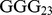. This may be attributed to the larger entropy of the partially formed structure containing 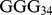, compared with that containing 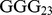. Physically, the entropy of the latter is lower in that it has two spatially close strands of length 7- and 8-nt, respectively, and the excluded volume effect between them lowers the structural entropy; in contrast, the former has a long unpaired strand of length 14-nt and a free nucleotide A24; the excluded volume effect between them is obviously minimal. Besides, the hairpin 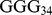 has lower enthalpy, according to two additional simulations performed for the two hairpins (Figure S16). Therefore, it is more likely that the early folding of the quadruplex starts from the hairpin 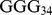.

The roles of non-native interactions in the folding process of G-DNA deserve further discussing. Before that, it is worth pointing out that in the research field of protein folding, non-native interactions are known to be important, particularly for the intrinsically disordered proteins. For example, Wang and colleagues studied the binding-induced folding of IA3, which is an intrinsically disordered protein that inhibits the yeast aspartic proteinase saccharopepsin by folding its own N-terminal residues into an alpha helix upon binding [40]. With their developed multi-scaled approach [41], [42], they found that the non-native interactions facilitate binding by reducing significantly the entropic search space in the landscape. Here in the folding of the G-DNA, the roles played by the non-native interactions were found to be similar. As described in the result section, the non-native interaction G9∶G4, together with the native A20∶T1, pull the first G-repeat close to the triplex and so that it will eventually dock on the triplex. Without these interactions, the first G-repeat may drift away and has to search in a much larger phase space. The above arguments can be easily verified by an experiment that measures the folding rates of the G-DNAs mutated on the corresponding nucleotides.

The structural formation and binding of metal ions are cooperative during the whole folding process. Physically, the effects of trapping of cations in the central channel of the quadruplex are twofold. First, the trapped cations compromise the strong negative charges of the backbone and facilitate their approaching to each other. Second, the metal ions are able to coordinate the O6 atoms of the nearby bases thus bridge the interactions between them. According to our simulations, the total number of bound ions increases monotonically from the intermediate II to V (Figure S10). In each intermediate, the formed base pairs need the binding of cations to strengthen their stabilities. For example, in the triplex structure of the intermediate-III, although G11, G15, and G23 are almost in their native position, they do not form stable base pairs according to Figure 2, as is correlated with the absence of the second 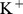 ions in the central channel. This feature is more clear in the intermediate-IV, where the above three nucleotides become even closer while the base pairs between them are still minimal (Figure 2 and 3), attributed to the same reason. Only after the G-DNA proceeds to the intermediate-V, the second ions is trapped in the central channel and then the surrounding base pairs become significantly stable. From another point of view, the trapping of cations also needs the formation of the local structures. This can be seen in the intermediate-IV, probably due to the lack of the protection from G5, the second 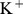 ion is able to leak out of the channel from the bottom (Figure 3) and thus cannot be trapped there stably (Figure 4). Therefore it is concluded that the folding and binding of ions are cooperative and mutually supporting each other.

The *syn/anti* reorientations are among the most important factors that affect the folding rate of G-DNAs. There are two different *syn/anti* reorientation dynamics according to our simulations. In general, the glycosidic bonds either stay at the correct configurations if the corresponding bases form native pairs with the others, or keep fluctuating if the bases are relatively free. In other words, in the correctly formed native structural elements defined by base pairs and backbone arrangements, wrong yet persistent *syn/anti* configurations are seldom observed, possible due to the steric inconsistence between local backbone arrangements and wrong *syn/anti* configurations. This feature is consistent with a previous work by Sugiyama *et al.*, who systematically studied all the possible loop conformations as well as the *syn/anti* arrangements for type-1 and type-2 quadruplexes using *ab initio* molecular dynamics and fragment molecular orbital calculations, and found that all the intermediate states leading to the native structure have correctly arranged *syn/anti* configurations [30]. Another support came from a recent simulation on G-DNAs by Šponer's group [43], where they concluded that for folding to a specific G-DNA topology in a single molecular event, the molecule must have an appropriate combination of *syn/anti* nucleotides, otherwise the likely result will be a misfolded structure. However, exceptions to the above pictures do exist according to our simulations. In the early folding intermediate-II and III, two nucleotides with wrong *syn/anti* configurations are observed although they form native pairs with the others (Figure 5). The exception may be explained by the outer positions of the nucleotides in the tertiary structure and the associated lacking of additional supports from nearby nucleotides or bound ions. Consistent with this argument, when the G-DNA folds to the intermediates-IV and further to V, more and more stable base pairs form and no wrong *syn/anti* configurations are observed any more.

Caution should be given regarding the limitations of the present simulation. First, although the current simulation detected intermediates only having less seriously wrong *syn/anti* patterns, mainly in the early folding stage and in the outer positioned nucleotides, it could not rule out the existence of other type of intermediates with most glycosidic bonds in wrong *syn/anti* configurations, since none of the replicas in BEMD was biased on the glycosidic bonds to enhance sampling on the relevant phase regions. Second, even if the existence of such intermediates could be ruled out by future computations, the *syn/anti* reorientations would still play an important role by significantly retarding the folding rate, since the molecule has to explore different combinations of *syn/anti* configurations in a much larger phase space to find the right bottle neck leading to the native states. Third, although the BEMD simulation was shown converged here, it should be noted that the convergence was subjected to the present setup of the applied CVs. In another word, the present simulation does not preclude that there is an orthogonal CV that samples intermediates not detected by the present setup. To finally confirm that the folding intermediates detected here are the only true intermediates would require significantly more expensive unbiased simulations and/or metadynamics with alternative and independent CVs. It is also worth mentioning that we had tested many different combinations of CVs and performed several times of BEMD simulations; the present four CVs were not chosen randomly. A much more large-scale unbiased simulation for the specific DNA is being prepared.

It is of particular interest to make a qualitatively comparison of the folding of quadruplexes with that of proteins. It seems that the former is more complex, since even for this small G-DNA of 24 nucleotides, four intermediate states have been identified. While in proteins, two-state folding is frequently observed for small globule proteins. Whether this is due to the particular topology of the quadruplex or the balance of interactions is not known yet. It is also interesting to characterize the main feature of the energy landscape of the G-quadruplexes and see if the energy funnel theory applies for these molecules. To this end, a topology-centered coarse-grained model of DNA quadruplex may be of help. The folding of quadruplexes is also complicated by the indispensable cooperation with metal ions, since the strong negative charges associated with the nucleotides have to be compensated by cations from solvents. According to our simulations, the metal ions progressively bind to the DNA as the quadruplex builds up, suggesting that the two process are cooperative. At last, the complexity is further increased by the involvement of the *syn/anti* reorientations of the glycosidic bonds, which increase the searching space and may also trap the G-DNA in some local minima.

**Figure 6:**
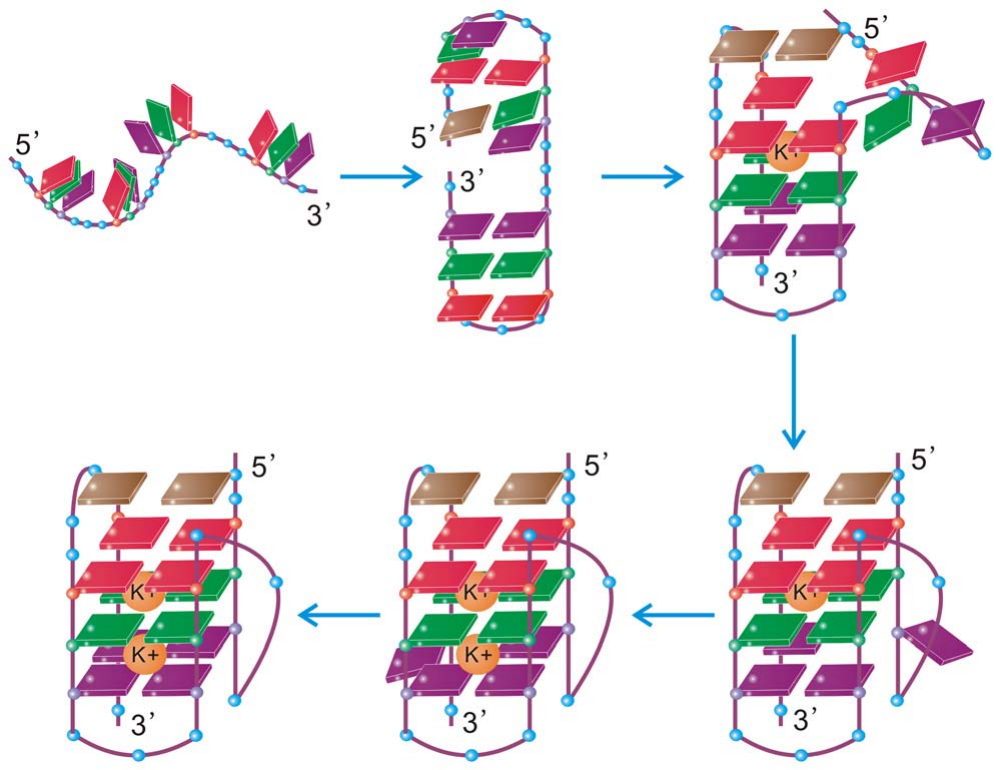
Schematic representation of the folding pathway proposed in this work.

In summary, enabled by the combined power of the bias-exchange metadynamics and large-scale conventional MD simulations, we studied the folding process of a hybrid-1 type human telomeric DNA G-quadruplex. We obtained for the first time its folding free energy landscape and identified several intermediates. Further analysis of these intermediates showed that the structure formation and metal ion binding are cooperative and mutually supporting each other. The roles of the *syn/anti* reorientations in the folding process were also investigated. It was found that the nucleotides already taking their native positions usually have correct *syn/anti* configurations. However, intermediates with wrong *syn/anti* configurations were also detected, particularly in the early folding stages. Based on the above results, we suggest a new atomistic folding picture for the G-DNA, as shown schematically in Figure 6 and described as follows. The G-DNA first forms a hairpin 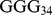 from the 3′-terminal, on which the second G-repeat docks, accompanied by the trapping of the first 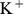 ion in the central channel. The result of the docking is a triplex. At this folding stage the first G-repeat is constrained nearby the triplex by both native and non-native interactions and fluctuating around the triplex. After the first G-repeat docks upon the triplex eventually, an incomplete quadruplex forms, and the reversal loop also basically forms at this stage. However, the second 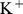 binding site in the central channel is yet to be occupied, and therefore the third G-tetrad is somewhat unstable. After another 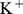 ion is trapped inside the channel, the whole quadruplex is strengthened and the folding is completed. We believe this is a more detailed and complete picture compared with previous ones, and it represents a step forward in understanding the folding of the hybrid-1 type G-DNA. The knowledge gained here may also provide insights into the structure formation processes of the other types of DNA G-quadruplexes.

## Materials and Methods

### System preparation

In the preparation of the simulation system, we solvated the PDB structure (2GKU) within a box of 6087 TIP3P water molecules and added 3 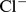 and 24 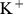 ions to achieve charge neutral and an equivalent 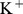 ion concentration of 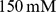. The amber99sb_parmbsc0 force field was used, which combined the amber99sb force field with new parmbsc0 nucleic acids torsions [44]. The ion parameters were taken to be their defaults values in the force field, which are 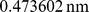 (sigma) and 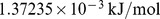 (epsilon) for 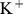, and 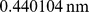 (sigma) and 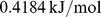 (epsilon) for 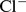. The electrostatic interaction was treated using PME with a cutoff of 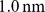. The same cutoff was used in the calculation of the van der Waals (VDW) interactions. All bonds were constrained using the LINCS algorithm and the MD time step was set to 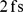. Berendsen algorithm was used for both temperature and pressure coupling. All simulations were performed with Gromacs (version:4.5.3) [45] and its plugin PLUMED (version 1.3) [46]. The whole system was first subjected to a minimization of 1000 steps and then an equilibrium run with a NPT ensemble at 1atm and 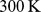 for 2 nanoseconds for a preparation of the initial structure. After that a long conventional MD simulation of length 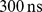 was performed started from this structure, in order to check the stability of the system setup and the native structure. It was found that during the simulation the fraction of native contacts (Q) was always higher than 0.92, all the native hydrogen bonds formed well, and the binding probabilities of 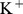 ions on 12 native sites were very close to unity, showing that the native structure is stable under the force field. The details of the trajectories are given in Figure S2.

### Bias-exchange metadynamics

The folding time of the specific DNA studied here is well beyond the timescale of traditional all-atom MD simulations. To overcome the barrier crossing problem, we adopted the bias-exchange metadynamics. In metadynamics, the overall external Gaussian potential acting on the system at time 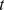 is given by
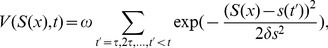
(1)where 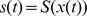 is the value taken by the Collective Variables at time 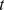, 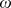 is the Gaussian height, 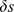 the Gaussian width, and 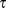 determines the frequency of adding Gaussian potentials. The basic assumption of metadynamics is that 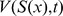 after a sufficiently long time provides an estimate of the underlying free energy:
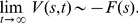
(2)


The bias-exchange metadynamics was run at 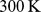 with four copies biased on four different CVs, respectively, as well as two neutral replicas without any bias. Four CVs included the fraction of native contacts formed between the 12 guanines (Q), the dRMSD of the backbone (C4* atoms) with respect to the native structure, the number of binding/contacts between 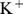 ions and the O6 atoms of the guanine bases (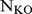), and the radius of the gyration (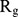). The parameters used for calculating these CVs were taken to be their default values in PLUMED [46]. The replicas were allowed to exchange their conformations and velocities periodically according to a metropolis-like criterion to further speed up the barrier crossing process. The criterion was given by
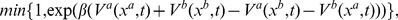
(3)where 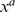 and 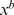 were the coordinates of walker 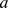 and 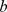, respectively, and 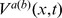 was the metadynamics potential acting on the walker 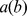.

Among all replicas five of them were started from the native structure, while one neutral replica was started from an extended structure obtained from unfolding simulations at a high temperature. During the BEMD run, the conformations and velocities of different replicas were exchanged periodically according to a metropolis criterion. The height of the repulsive Gaussian potentials were 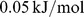 and their widths were set to 2.5/130, 0.02 nm, 0.5, and 0.2 nm, for Q, dRMSD, 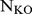, and 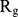, respectively. Note that the above number 130 is the total number of native contacts. The deposition rate of the Gaussian potentials is 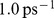. The attempting frequency for replica exchanges was set to 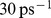. The overall simulation time of the metadynamics was 4.2 microseconds, with each replica lasting for 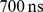. The convergence of the calculation was shown in Figure S3 and discussed in the main text.

### Conventional MD simulations

To reveal the structures of the intermediates as well as their dynamics, we resorted to additional conventional MD simulations. For each of the four intermediates, we randomly selected three structures in the largest cluster obtained by a clustering analysis, and then for each structure we performed 100 ns MD simulations. The system setup, the force field and the parameters for running conventional simulations were the same as described above. There were in total 12 trajectories and the overall simulation time was 1.2 µs. The details of the trajectories are given in the Figure S5, S6, S7, S8, S9, S10, S11, S12, S13, S14.

### The clustering algorithm

We adopted a simple algorithm to cluster the conformations obtained in MD simulations. We compared the 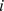-th frame in the trajectories with the representative structures of the clusters obtained previously one by one; if a dRMSD smaller than a threshold was detected, the 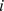-th frame was deemed belonging to the corresponding cluster; if the 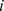-th frame did not belong to any existing clusters, it was assumed to be the representative structure of a new cluster. The threshold for determining if two structures belonged to the same cluster was set to be 0.3 nm in all analysis except in the clustering of high-temperature unfolding trajectories described later, where the threshold is set to be 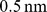.

### Unfolding simulations

Unfolding simulations were started from the native structure and performed at 1atm and 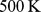 to enhance the barrier crossing events. The system setup was the same as described above. In total 10 such simulations were performed with each lasting for 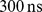. After the simulations were finished, we performed clustering analysis to get the unfolding pathways, as shown in Figure S15.

### Additional MD simulations for two hairpins

We performed two additional MD simulations for two hairpins 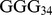 and 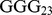 to compare their stability. For the hairpin 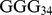, we chopped the hairpin fragment from the native structure starting from A14 to A24 and deleted the other nucleotides; the remaining length was 11-nt. Similarly, for 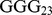 we retained the structure from A8 to T18; the remaining length was also 11-nt. For each hairpin, we solved it in explicit waters and added ions to achieve charge neutral and the same 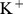 concentration. The obtained two systems had the same number of 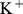 ions and almost the same number of water molecules (3,928 versus 3,930). Both hairpins were restrained to their native structures with weak harmonic potentials. The MD simulations were performed at 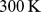 and 1atm for 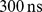 each. After the simulations were finished, we calculated the enthalpy of the hairpins by excluding the restraint energy, as shown in Figure S16.

## Supporting Information

Figure S1
**Native structure of the G-DNA.** (A) The native structure of the 24-nt DNA sequence 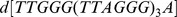. It has a (3+1) G-quadruplex topology in which three strands are oriented in one direction and one in the opposite direction. From the top down, the three G-tetrads are colored red, green, and purple, respectively. The 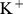 ions are plotted as orange spheres. (B) The corresponding schematic representation of the native structure, colored in the same code as (A).(TIF)

Figure S2
**MD results for the native structure.** (A) The evolution of the fraction of native contact Q as a function of time, calculated from a MD simulation starting from the native structure. This is to test the stability of the system setup and of the native structure. The MD trajectory lasts for 300 ns and is very stable, indicated by the close-to-unity values throughout the whole simulation. (B) The hydrogen bond map averaged on the conformations obtained in the above 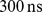 MD run, with the formation probabilities indicated by the color scale. (C) The 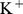 ion binding probabilities on each nucleotide calculated from the same simulation. The total number of bound 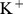 ions is close to 3, with two ions trapped inside the central channel of the quadruplex and the third distributed almost evenly on all nucleotides.(TIF)

Figure S3
**Convergence tests for the bias-exchange metadynamics.** (A) Random walk in their respective CV spaces calculated for four biased replicas. (B) The free energy landscapes (FELs) calculated after 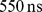, 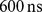, 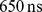, and 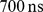 runs of BEMD; the data for making the calculations was taken solely from one neutral replica. (C) and (D) are the zoomed FELs calculated at 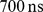 from two neutral replicas, respectively. (E) The number of successfully exchanged events as a function of time for six replicas. The average exchange probabilities are 5.7%, 4.8%, 3.9%, 4.2%, 21.7%, 21.9%, respectively. The lower probabilities in the first four replicas are expected since the replicas have very different energies due to the different biases applied. The curves are almost linear as a function of time, suggesting that a steady exchange rate is maintained throughout the whole simulation.(TIF)

Figure S4
**Results for the first basin of attraction, i.e., the denatured states.** (A) The hydrogen bond map. The hydrogen bonds indicated by the white arrows are the non-native ones. (B) The 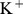 ion binding probabilities on each nucleotide. The red, green, and purple histograms correspond to the binding probabilities on the three G-tetrads, respectively. The color code is the same as in Figure 1 in the main text and as in Figure S1. (C) The representative structures from the largest three clusters, respectively. (D) The normalized populations of the largest twenty clusters. Note that figures (A)–(D) were calculated based on the structures collected in BEMD, not in conventional MD simulations, since this basin is highly heterogeneous and cannot be covered by several conventional MD trajectories.(TIF)

Figure S5 **Conventional MD trajectories calculated for the intermediate-II.** Different columns correspond to different simulations while different rows give the time evolution of different parameters. At the bottom, the initial and the last structures at the end of simulations are shown.
(TIF)

Figure S6
**Conventional MD trajectories calculated for the intermediate-III.** For detailed caption, see Figure S5. The first two columns correspond to a docking of the first G-repeat on the triplex, while the last column corresponds to a flanking motion of the first G-repeat with respect to the triplex. The docking is reflected by a sudden increase of the Q and 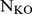 values, and can be seen more clearly in Figure S9.(TIF)

Figure S7 **Conventional MD trajectories calculated for the intermediate-IV.** For detailed caption, see Figure S5.
(TIF)

Figure S8
**Conventional MD trajectories calculated for the intermediate-V.** For detailed caption, see Figure S5. The first and last columns show two direct folding events to the native basin of attraction, reflected by a sudden jump to higher regions of Q and 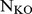 and the extremely small fluctuation that follows.(TIF)

Figure S9 **The projection of 12 conventional MD trajectories on the FEL.** The trajectories started from the intermediate-II, III, IV and V are colored red, green, brown, and blue, respectively. Two folding events from the intermediate-III to the intermediate-IV can be seen.
(TIF)

Figure S10
**The total number of bound** 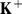 **ions calculated for each basin/intermediate.** Note that the overall number of 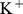 ions in the native state is close to 3.(TIF)

Figure S11 **The** ***syn/anti*** **isomerization of the glycosidic bonds as a function of time calculated for the intermediate-II.** The trajectories were obtained from the same simulations shown in Figure S5, S6, S7, S8. The nucleotides belonging to the same G-tetrads are plotted in the same row while that belonging to the same G-repeats in the same column.
(TIF)

Figure S12 **The** ***syn/anti*** **isomerization of the glycosidic bonds as a function of time calculated for the intermediate-III.** Similar to Figure S11.
(TIF)

Figure S13 **The** ***syn/anti*** **isomerization of the glycosidic bonds as a function of time calculated for the intermediate-IV.** Similar to Figure S11.
(TIF)

Figure S14 **The** ***syn/anti*** **isomerization of the glycosidic bonds as a function of time calculated for the intermediate-V.** Similar to Figure S11.
(TIF)

Figure S15 **The unfolding pathways.** The structures were obtained by a clustering analysis of 10 unfolding trajectories. The numbers beside the arrows indicate the number of trajectories going through that pathway.
(TIF)

Figure S16
**The enthalpy difference between two hairpins** 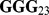 **and** 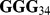 **.** The two structures shown at the top are the last frames of the simulations. The enthalpy includes both contribution from DNA and water molecules.(TIF)

Video S1 **A movie showing the dynamics of the intermediate-II.**
(AVI)

Video S2
**A movie showing the dynamics of the intermediate-III and the docking of** 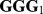 **on the triplex.**
(AVI)

Video S3
**A movie showing the flanking motion of the** 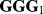 **with respect to the triplex in the intermediate-III.**
(AVI)
