# Supplementary figures and images for: Atomistic Picture for the Folding Pathway of a Hybrid-1 Type Human Telomeric DNA G-quadruplex
Source: PLoS Comput Biol. 2014 Apr 10;10(4):e1003562. doi: 10.1371/journal.pcbi.1003562 (PMC3983051; doi:10.1371/journal.pcbi.1003562)

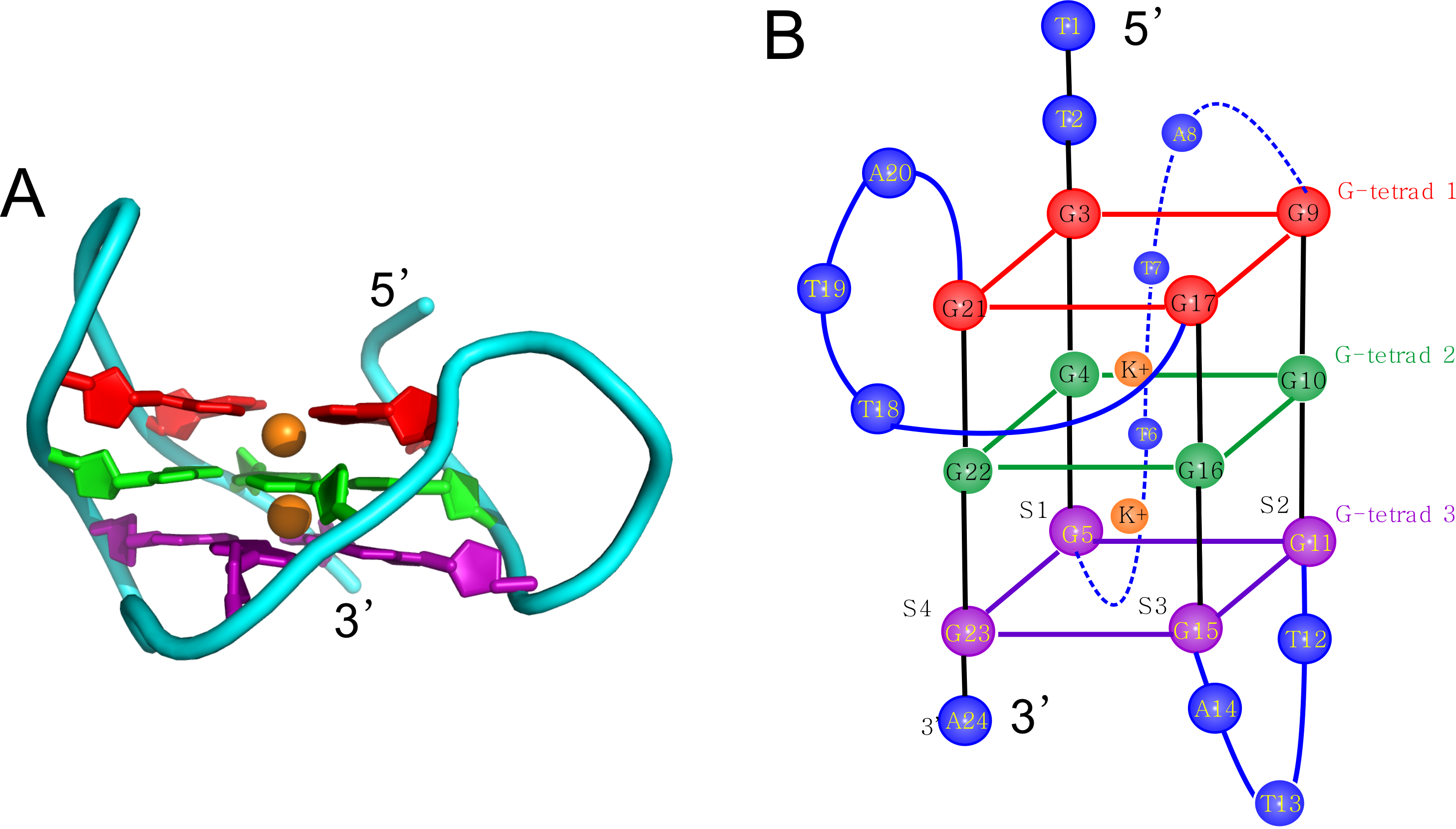

Supplement: Figure S1 — Native structure of the G-DNA. (A) The native structure of the 24-nt DNA sequence . It has a (3+1) G-quadruplex topology in which three strands are oriented in one direction and one in the opposite direction. From the top down, the three G-tetrads are colored red, green, and purple, respectively. The ions are plotted as orange spheres. (B) The corresponding schematic representation of the native structure, colored in the same code as (A). (TIF) [file pcbi.1003562.s001.tif]

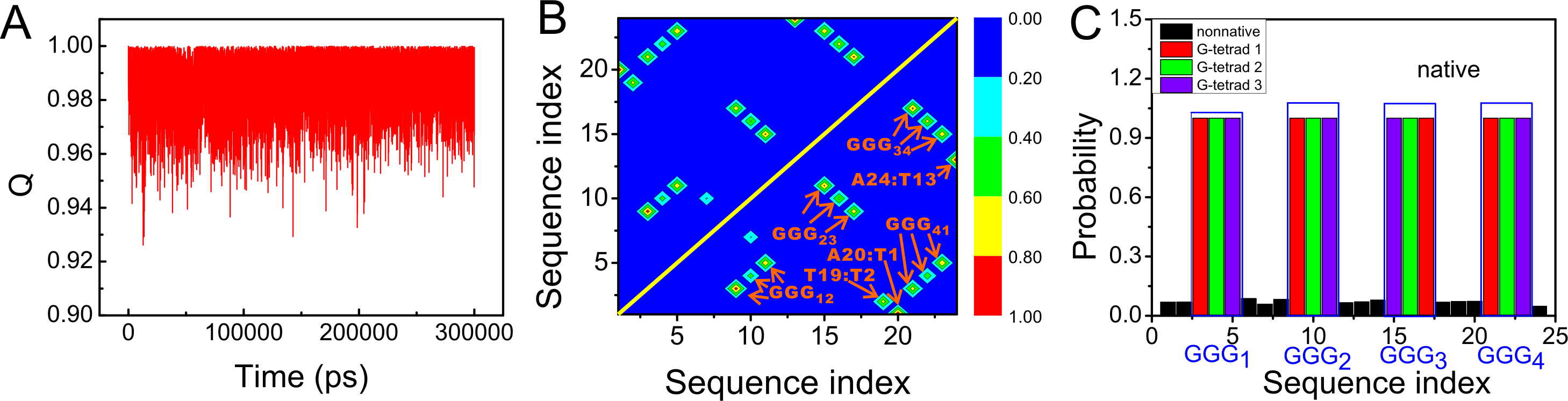

Supplement: Figure S2 — MD results for the native structure. (A) The evolution of the fraction of native contact Q as a function of time, calculated from a MD simulation starting from the native structure. This is to test the stability of the system setup and of the native structure. The MD trajectory lasts for 300 ns and is very stable, indicated by the close-to-unity values throughout the whole simulation. (B) The hydrogen bond map averaged on the conformations obtained in the above MD run, with the formation probabilities indicated by the color scale. (C) The ion binding probabilities on each nucleotide calculated from the same simulation. The total number of bound ions is close to 3, with two ions trapped inside the central channel of the quadruplex and the third distributed almost evenly on all nucleotides. (TIF) [file pcbi.1003562.s002.tif]

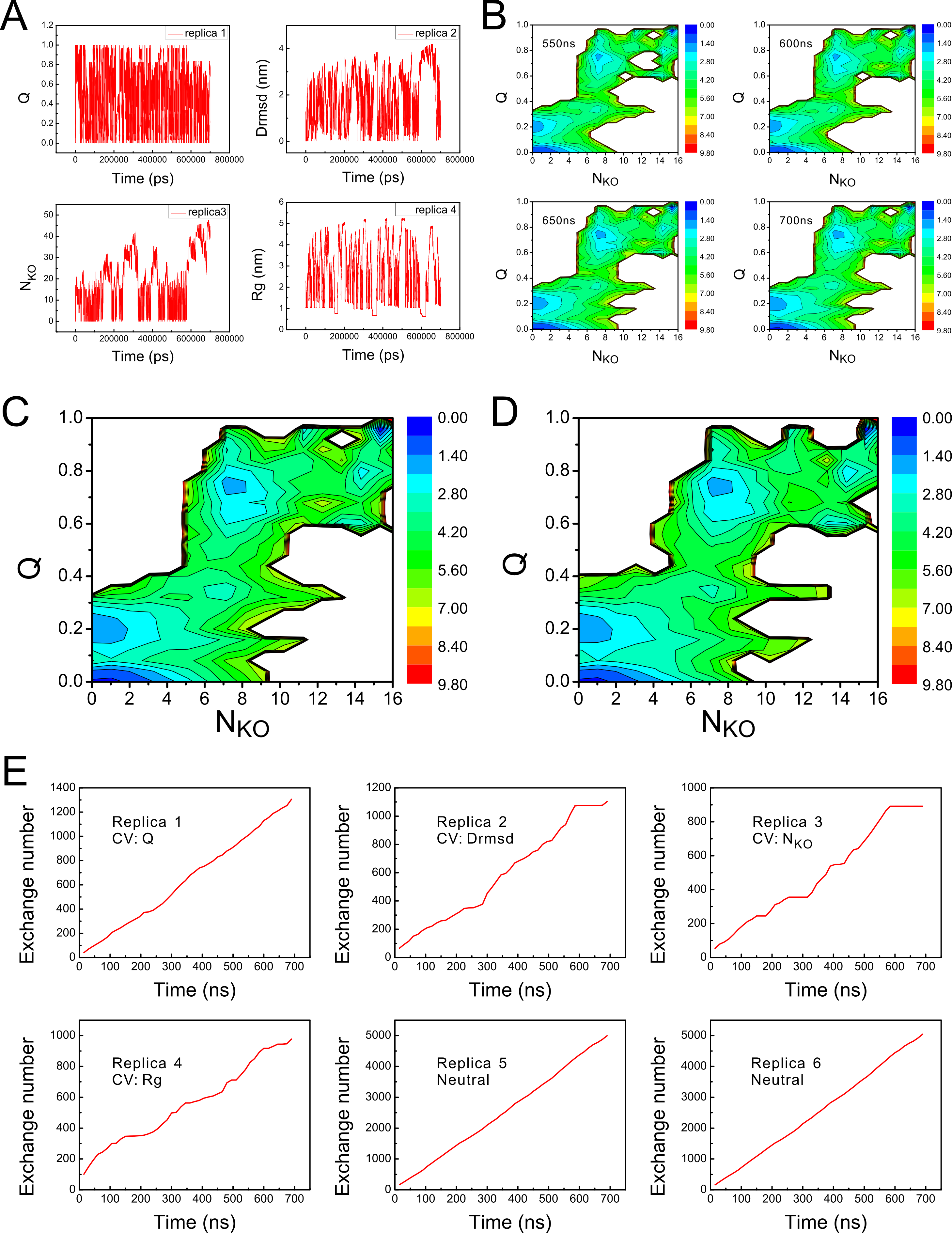

Supplement: Figure S3 — Convergence tests for the bias-exchange metadynamics. (A) Random walk in their respective CV spaces calculated for four biased replicas. (B) The free energy landscapes (FELs) calculated after , , , and runs of BEMD; the data for making the calculations was taken solely from one neutral replica. (C) and (D) are the zoomed FELs calculated at from two neutral replicas, respectively. (E) The number of successfully exchanged events as a function of time for six replicas. The average exchange probabilities are 5.7%, 4.8%, 3.9%, 4.2%, 21.7%, 21.9%, respectively. The lower probabilities in the first four replicas are expected since the replicas have very different energies due to the different biases applied. The curves are almost linear as a function of time, suggesting that a steady exchange rate is maintained throughout the whole simulation. (TIF) [file pcbi.1003562.s003.tif]

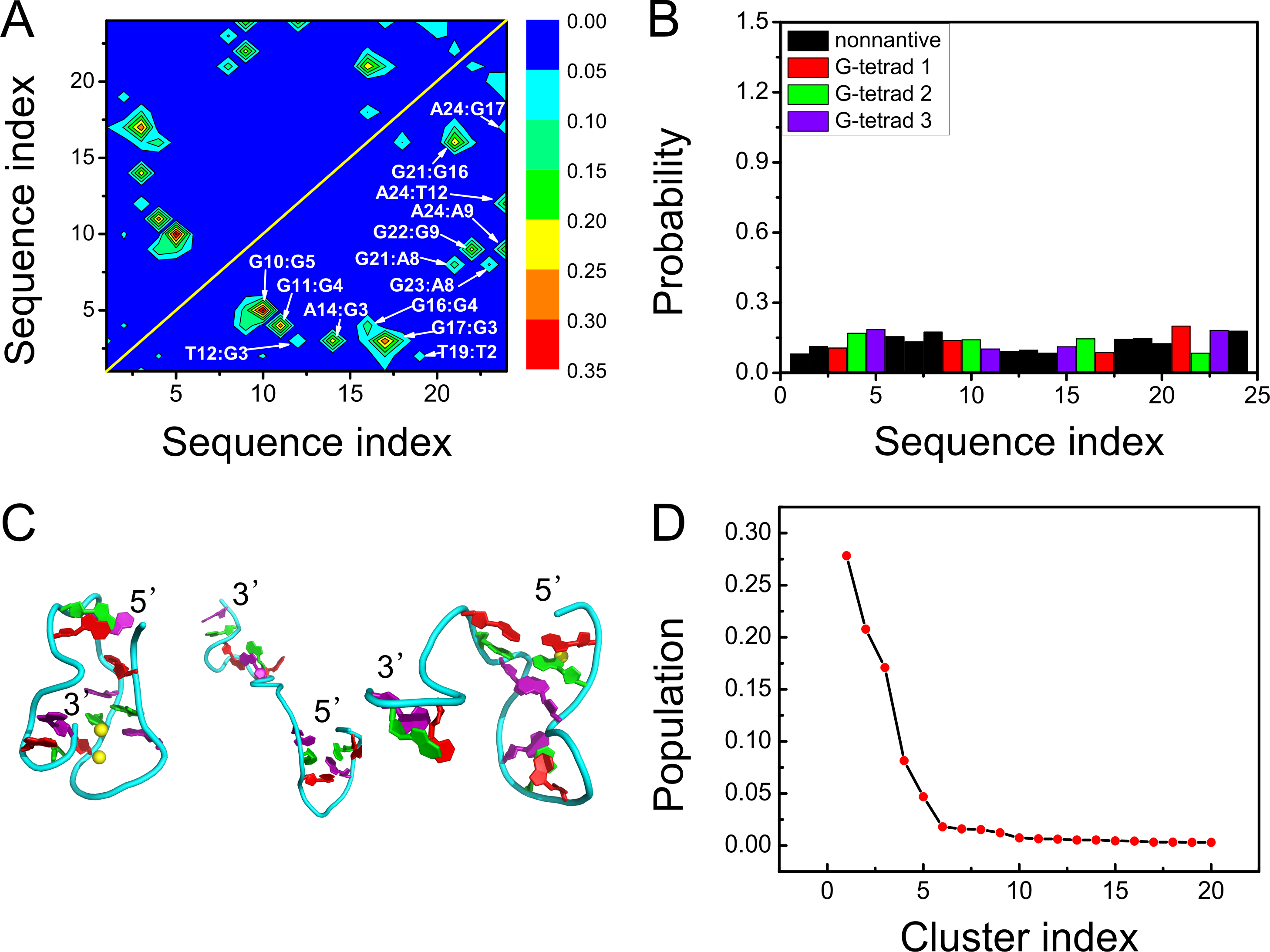

Supplement: Figure S4 — Results for the first basin of attraction, i.e., the denatured states. (A) The hydrogen bond map. The hydrogen bonds indicated by the white arrows are the non-native ones. (B) The ion binding probabilities on each nucleotide. The red, green, and purple histograms correspond to the binding probabilities on the three G-tetrads, respectively. The color code is the same as in Figure 1 in the main text and as in Figure S1. (C) The representative structures from the largest three clusters, respectively. (D) The normalized populations of the largest twenty clusters. Note that figures (A)–(D) were calculated based on the structures collected in BEMD, not in conventional MD simulations, since this basin is highly heterogeneous and cannot be covered by several conventional MD trajectories. (TIF) [file pcbi.1003562.s004.tif]

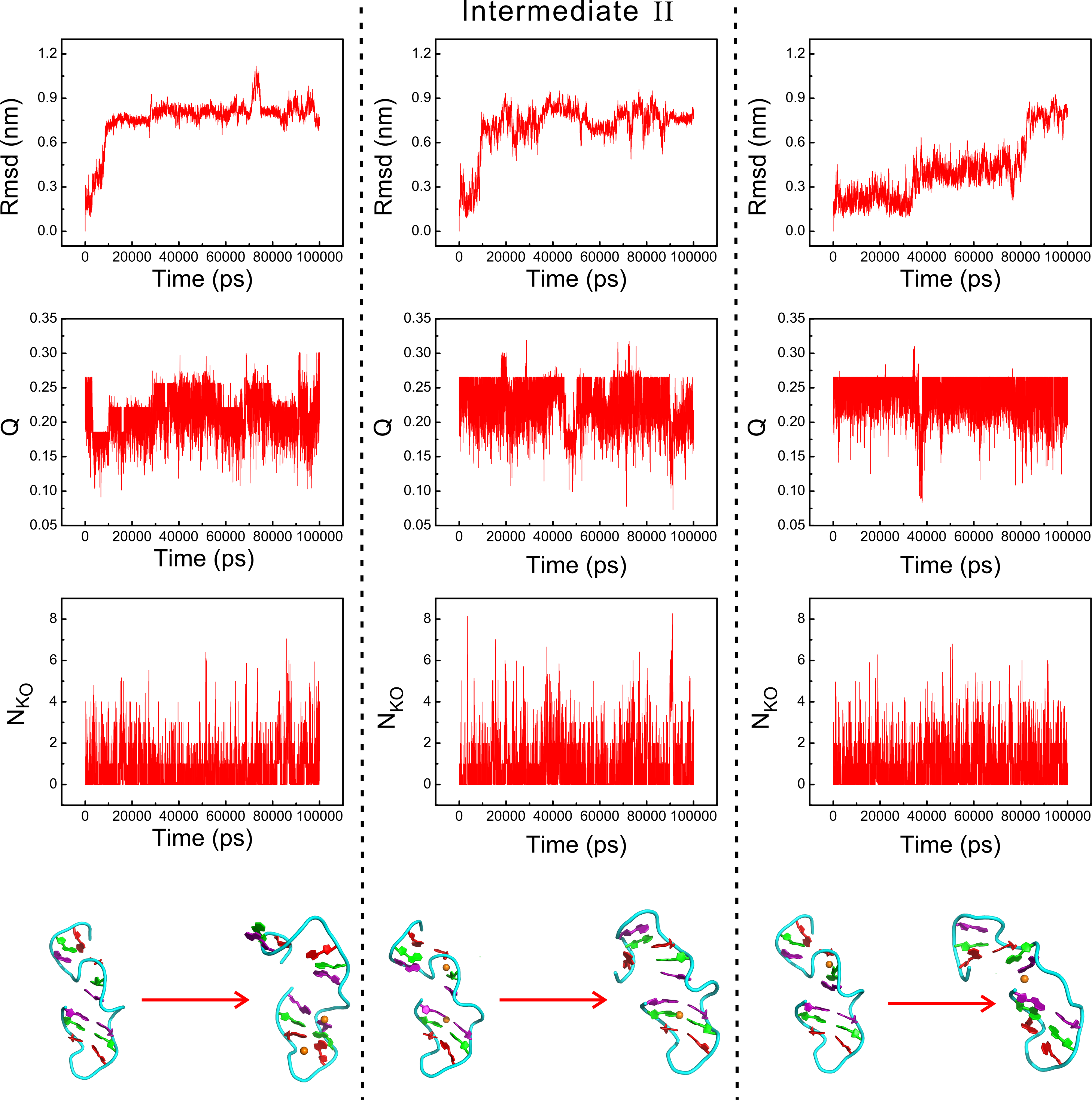

Supplement: Figure S5 — Conventional MD trajectories calculated for the intermediate-II. Different columns correspond to different simulations while different rows give the time evolution of different parameters. At the bottom, the initial and the last structures at the end of simulations are shown. (TIF) [file pcbi.1003562.s005.tif]

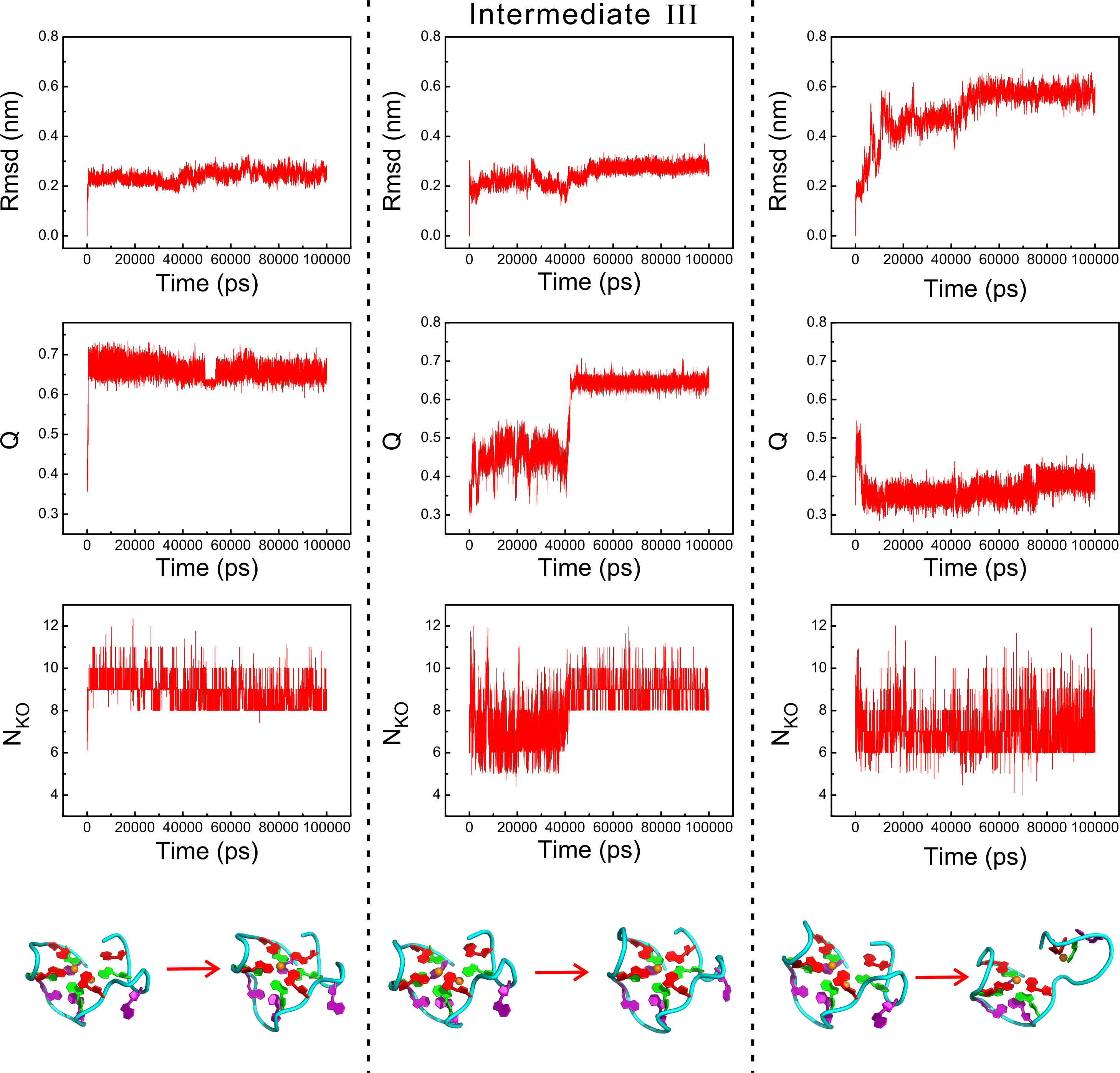

Supplement: Figure S6 — Conventional MD trajectories calculated for the intermediate-III. For detailed caption, see Figure S5. The first two columns correspond to a docking of the first G-repeat on the triplex, while the last column corresponds to a flanking motion of the first G-repeat with respect to the triplex. The docking is reflected by a sudden increase of the Q and values, and can be seen more clearly in Figure S9. (TIF) [file pcbi.1003562.s006.tif]

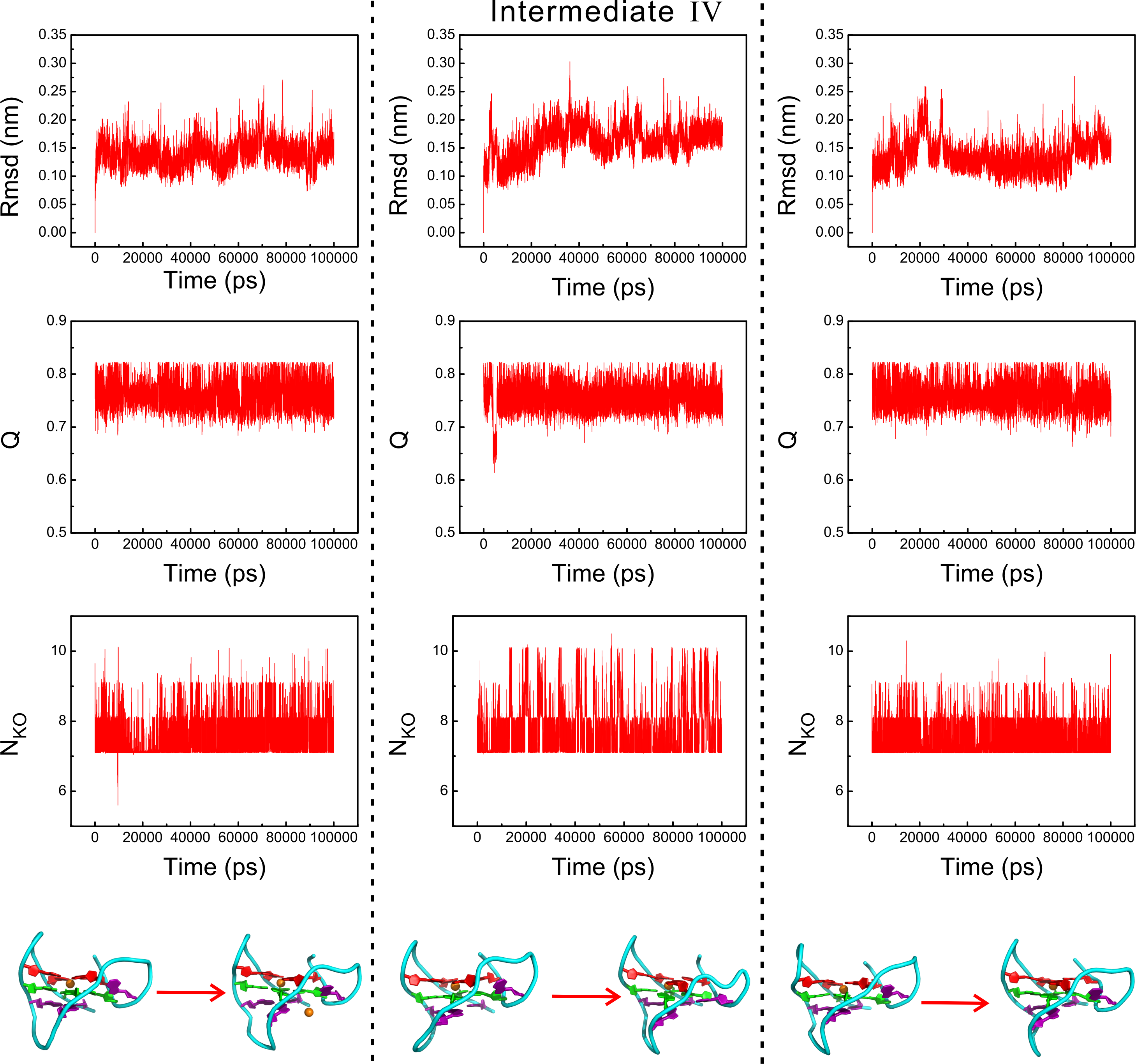

Supplement: Figure S7 — Conventional MD trajectories calculated for the intermediate-IV. For detailed caption, see Figure S5. (TIF) [file pcbi.1003562.s007.tif]

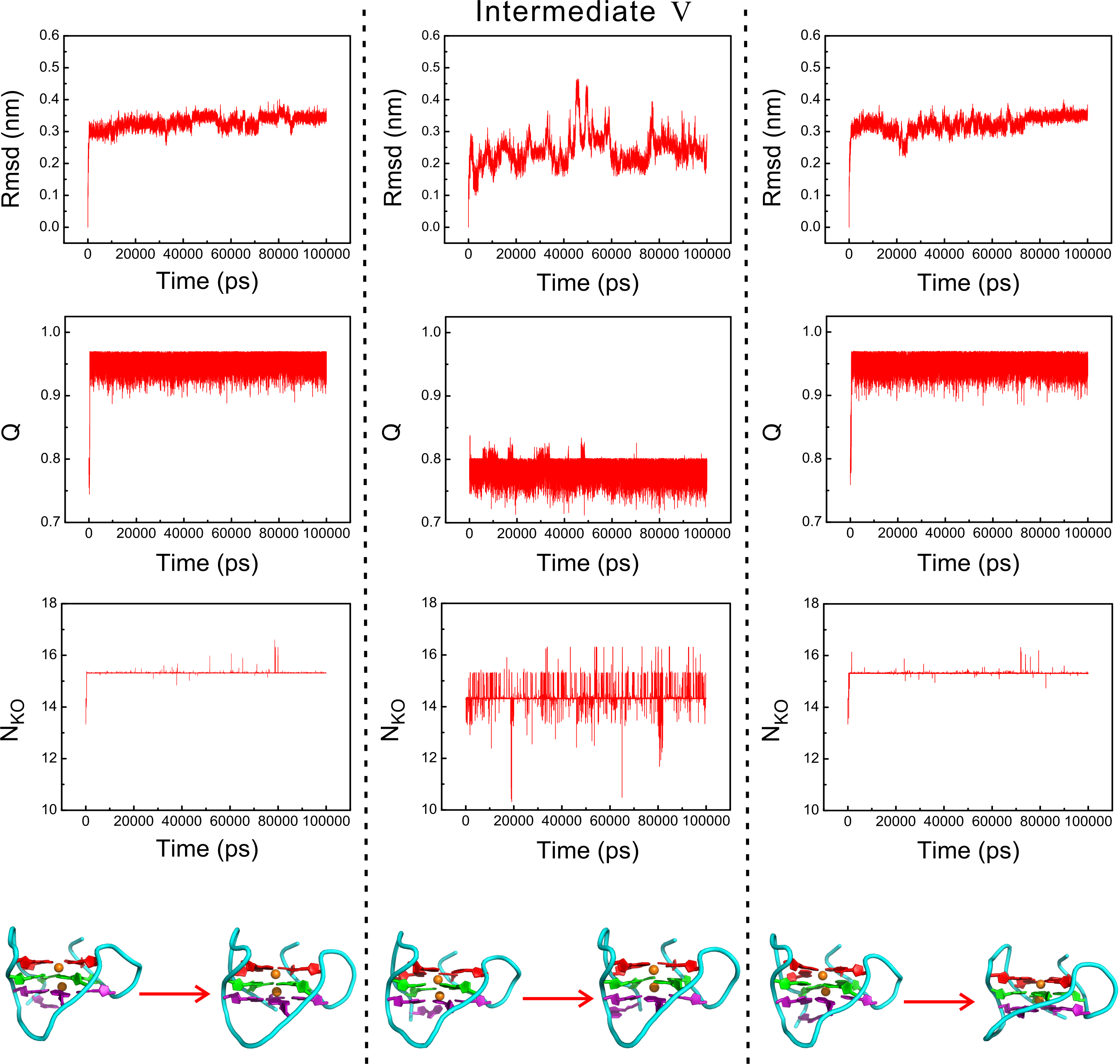

Supplement: Figure S8 — Conventional MD trajectories calculated for the intermediate-V. For detailed caption, see Figure S5. The first and last columns show two direct folding events to the native basin of attraction, reflected by a sudden jump to higher regions of Q and and the extremely small fluctuation that follows. (TIF) [file pcbi.1003562.s008.tif]

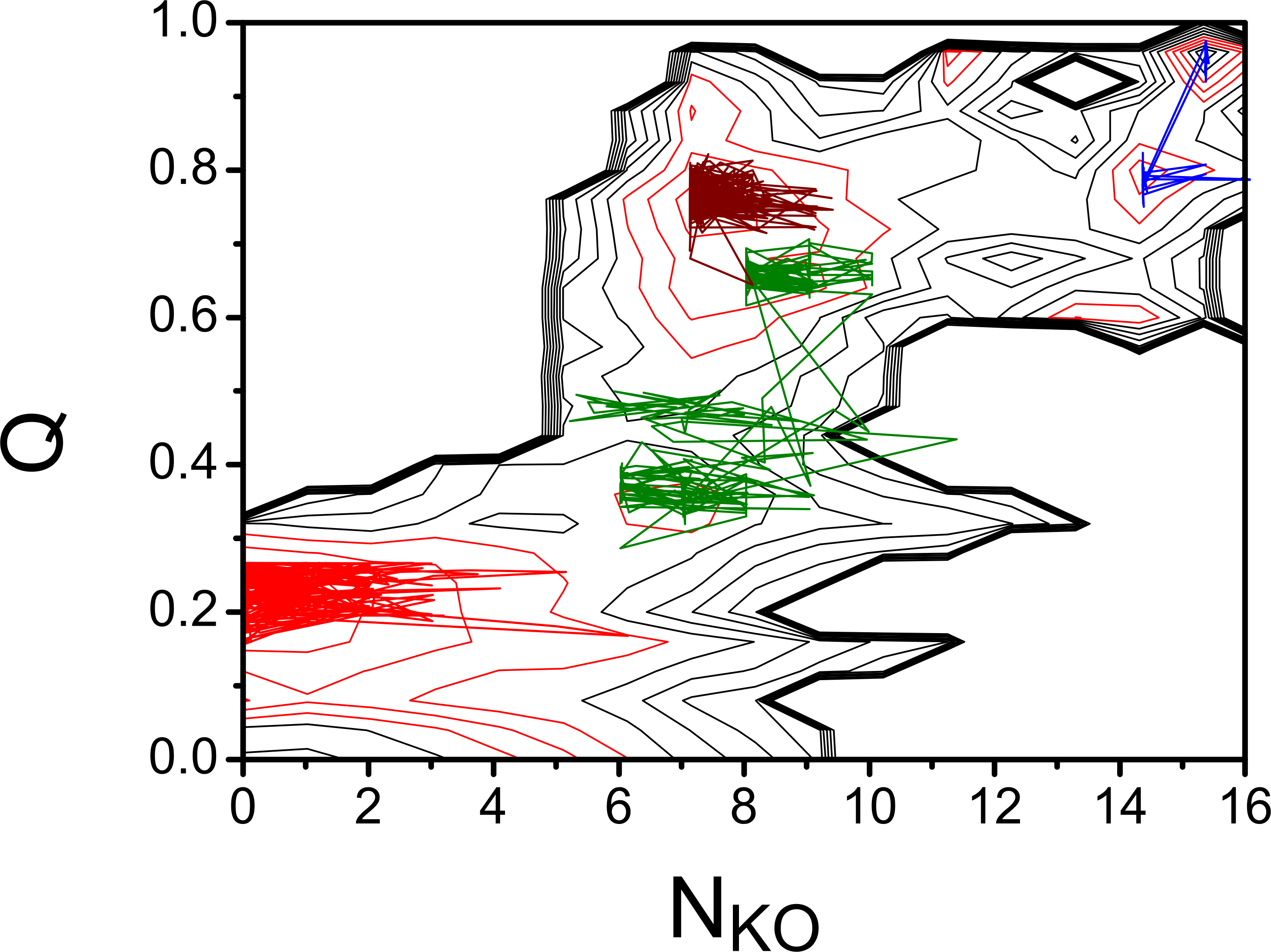

Supplement: Figure S9 — The projection of 12 conventional MD trajectories on the FEL. The trajectories started from the intermediate-II, III, IV and V are colored red, green, brown, and blue, respectively. Two folding events from the intermediate-III to the intermediate-IV can be seen. (TIF) [file pcbi.1003562.s009.tif]

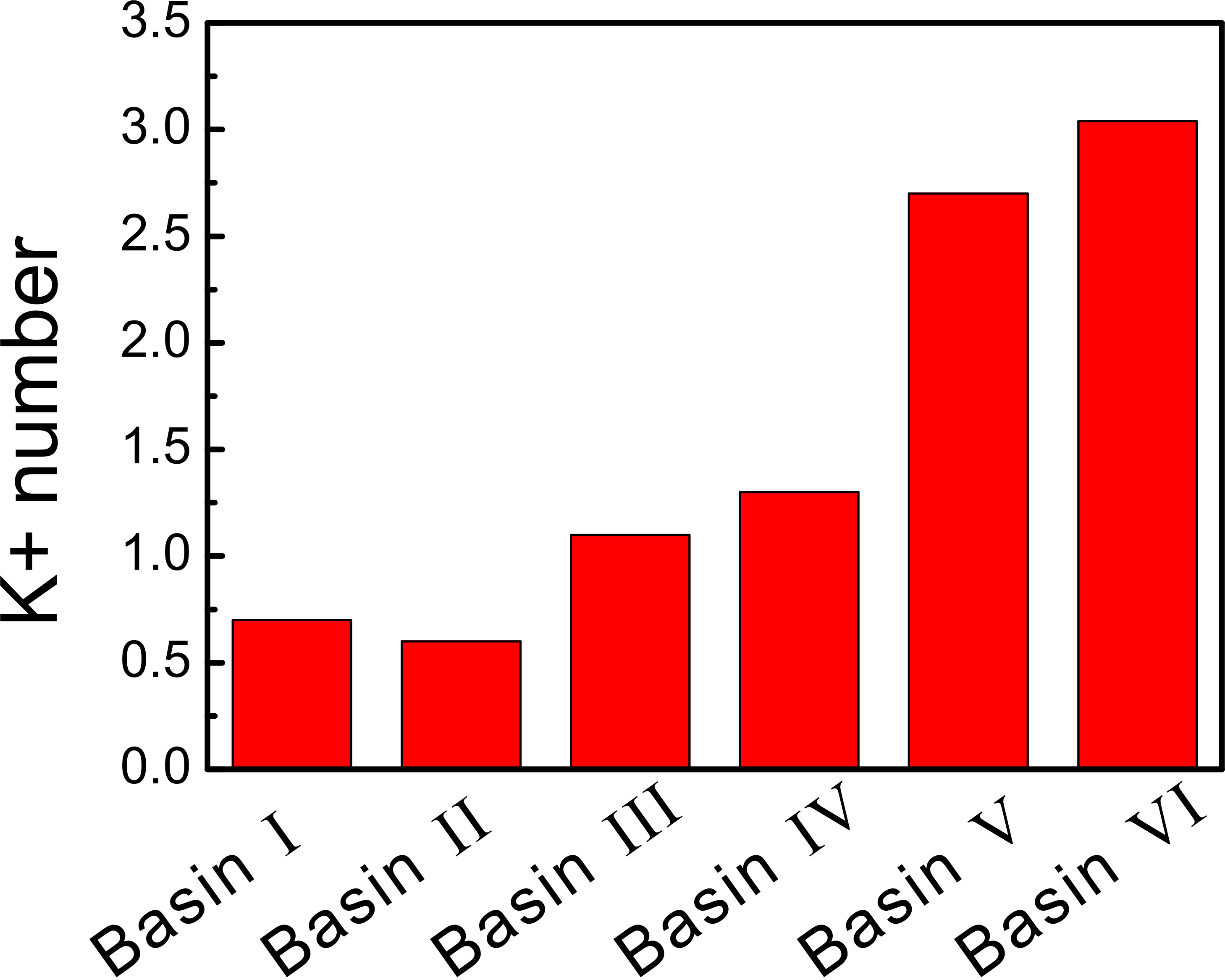

Supplement: Figure S10 — The total number of bound ions calculated for each basin/intermediate. Note that the overall number of ions in the native state is close to 3. (TIF) [file pcbi.1003562.s010.tif]

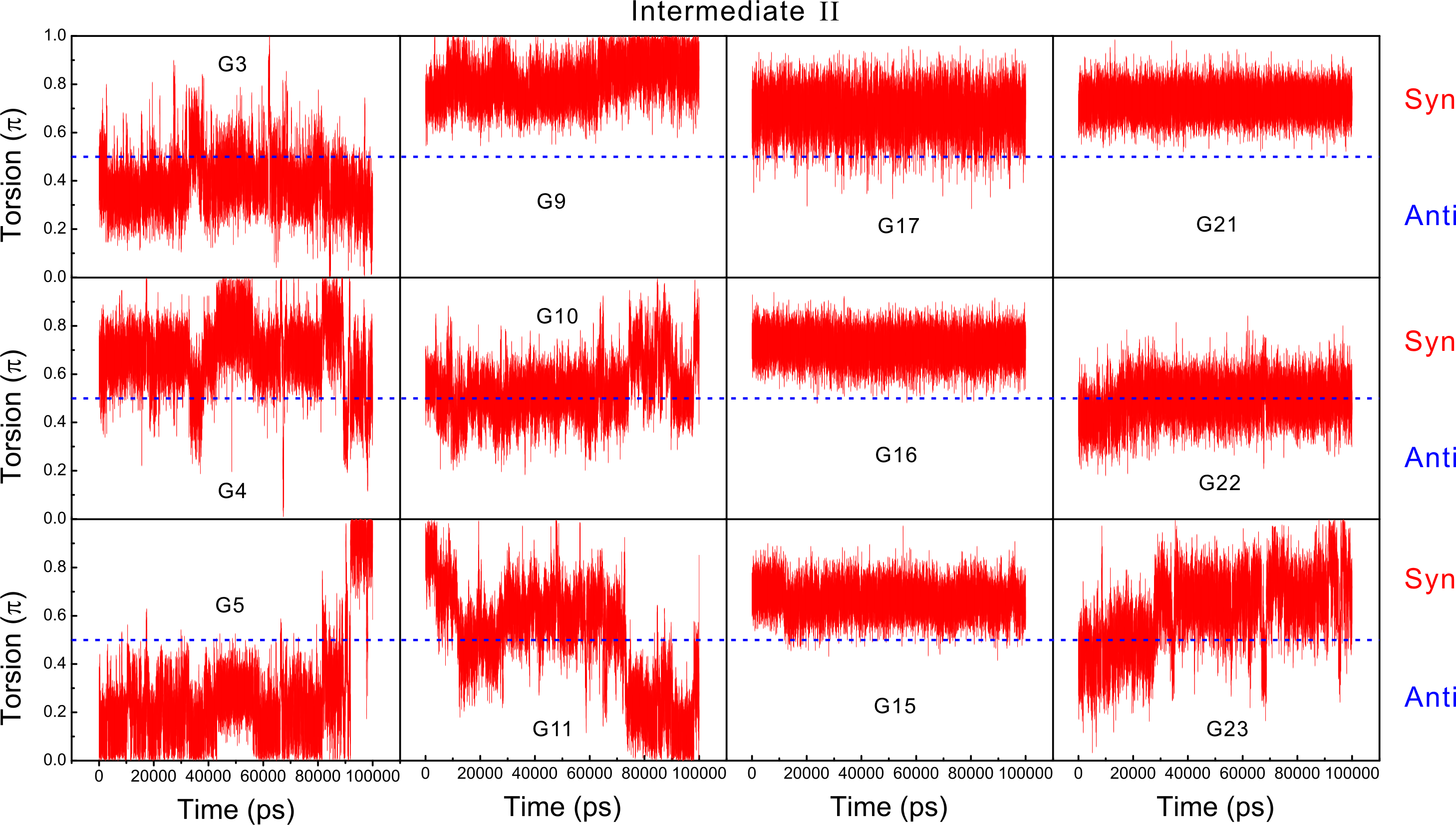

Supplement: Figure S11 — The syn/anti isomerization of the glycosidic bonds as a function of time calculated for the intermediate-II. The trajectories were obtained from the same simulations shown in Figure S5, S6, S7, S8. The nucleotides belonging to the same G-tetrads are plotted in the same row while that belonging to the same G-repeats in the same column. (TIF) [file pcbi.1003562.s011.tif]

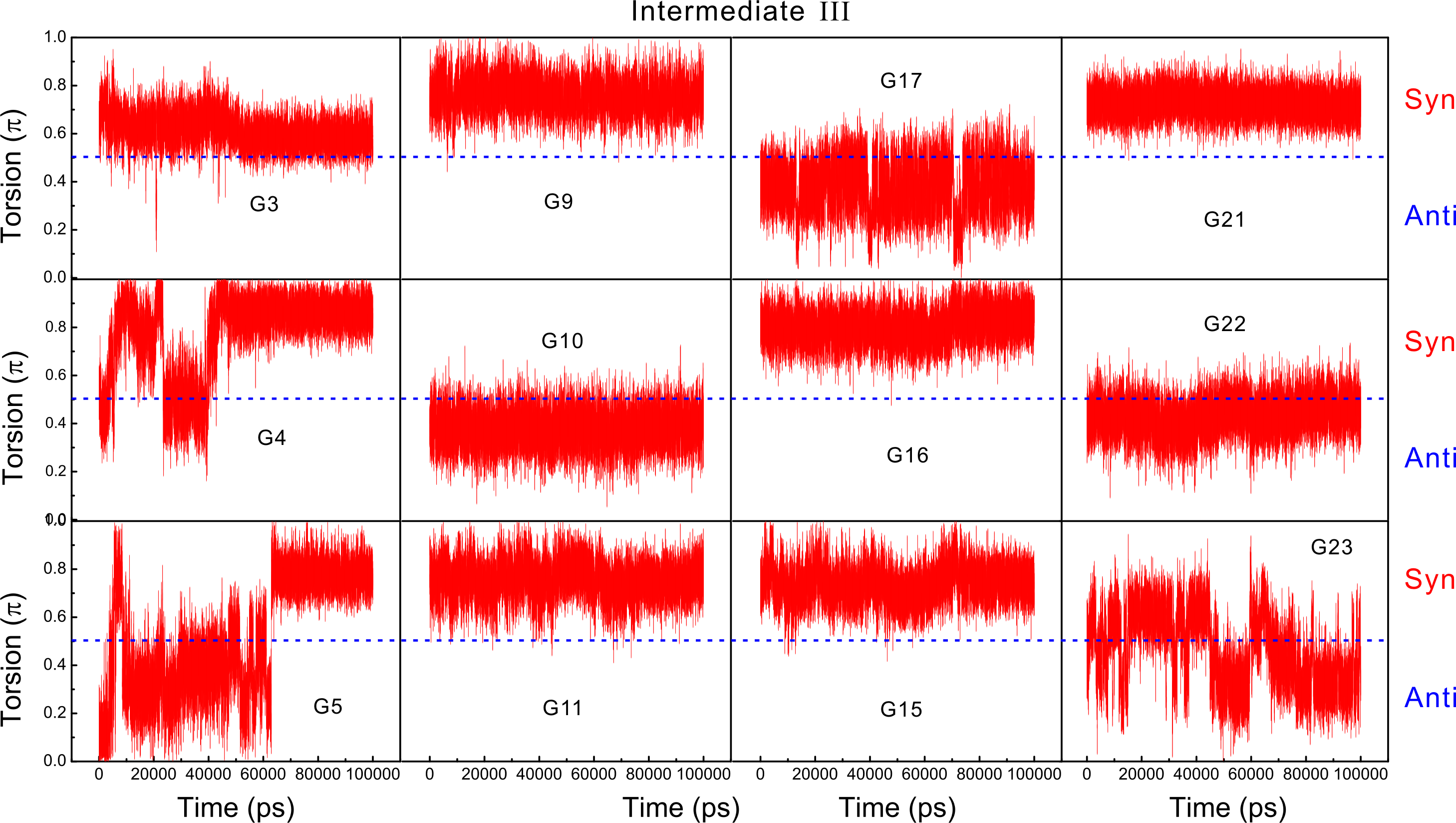

Supplement: Figure S12 — The syn/anti isomerization of the glycosidic bonds as a function of time calculated for the intermediate-III. Similar to Figure S11. (TIF) [file pcbi.1003562.s012.tif]

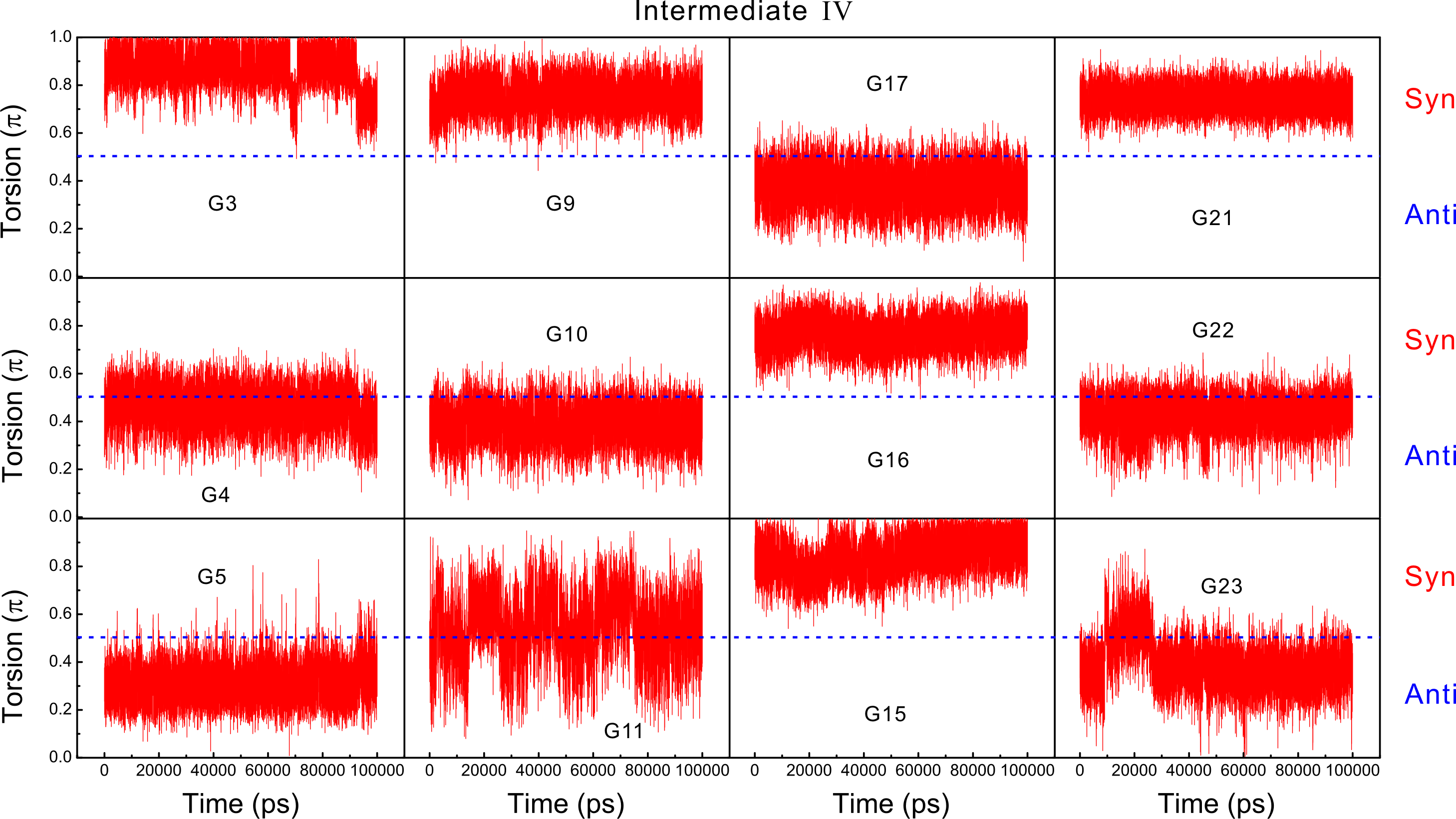

Supplement: Figure S13 — The syn/anti isomerization of the glycosidic bonds as a function of time calculated for the intermediate-IV. Similar to Figure S11. (TIF) [file pcbi.1003562.s013.tif]

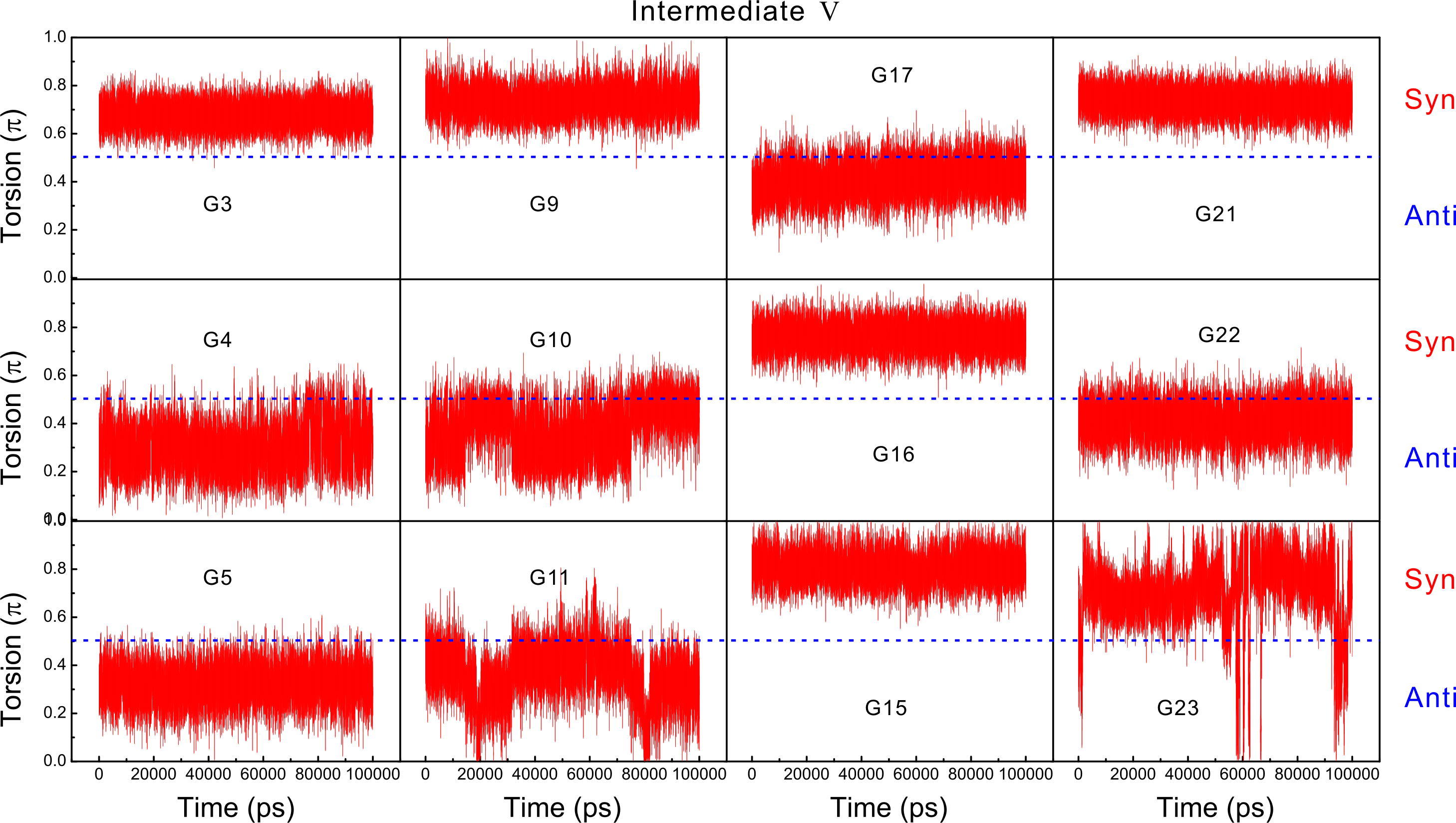

Supplement: Figure S14 — The syn/anti isomerization of the glycosidic bonds as a function of time calculated for the intermediate-V. Similar to Figure S11. (TIF) [file pcbi.1003562.s014.tif]

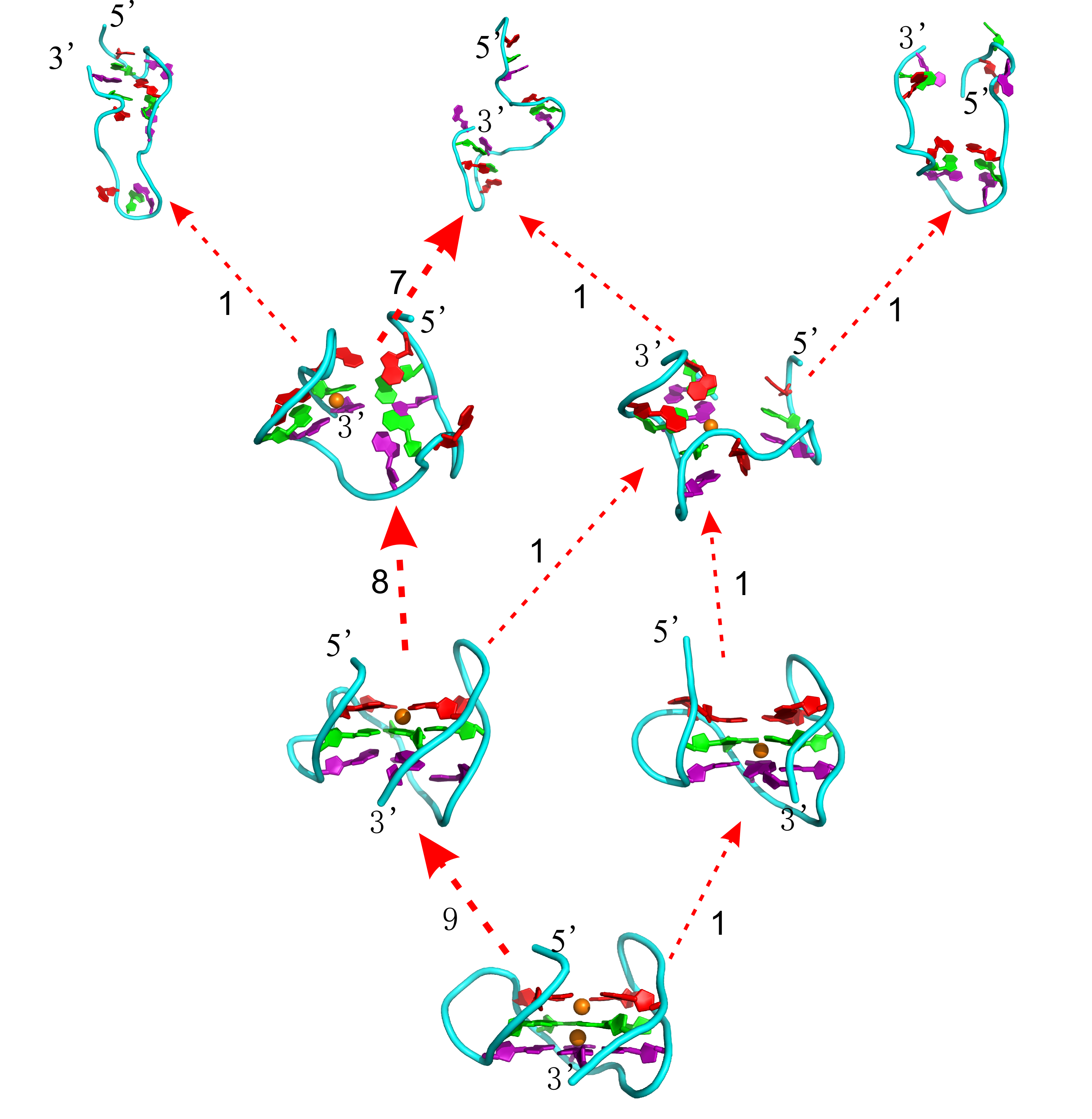

Supplement: Figure S15 — The unfolding pathways. The structures were obtained by a clustering analysis of 10 unfolding trajectories. The numbers beside the arrows indicate the number of trajectories going through that pathway. (TIF) [file pcbi.1003562.s015.tif]

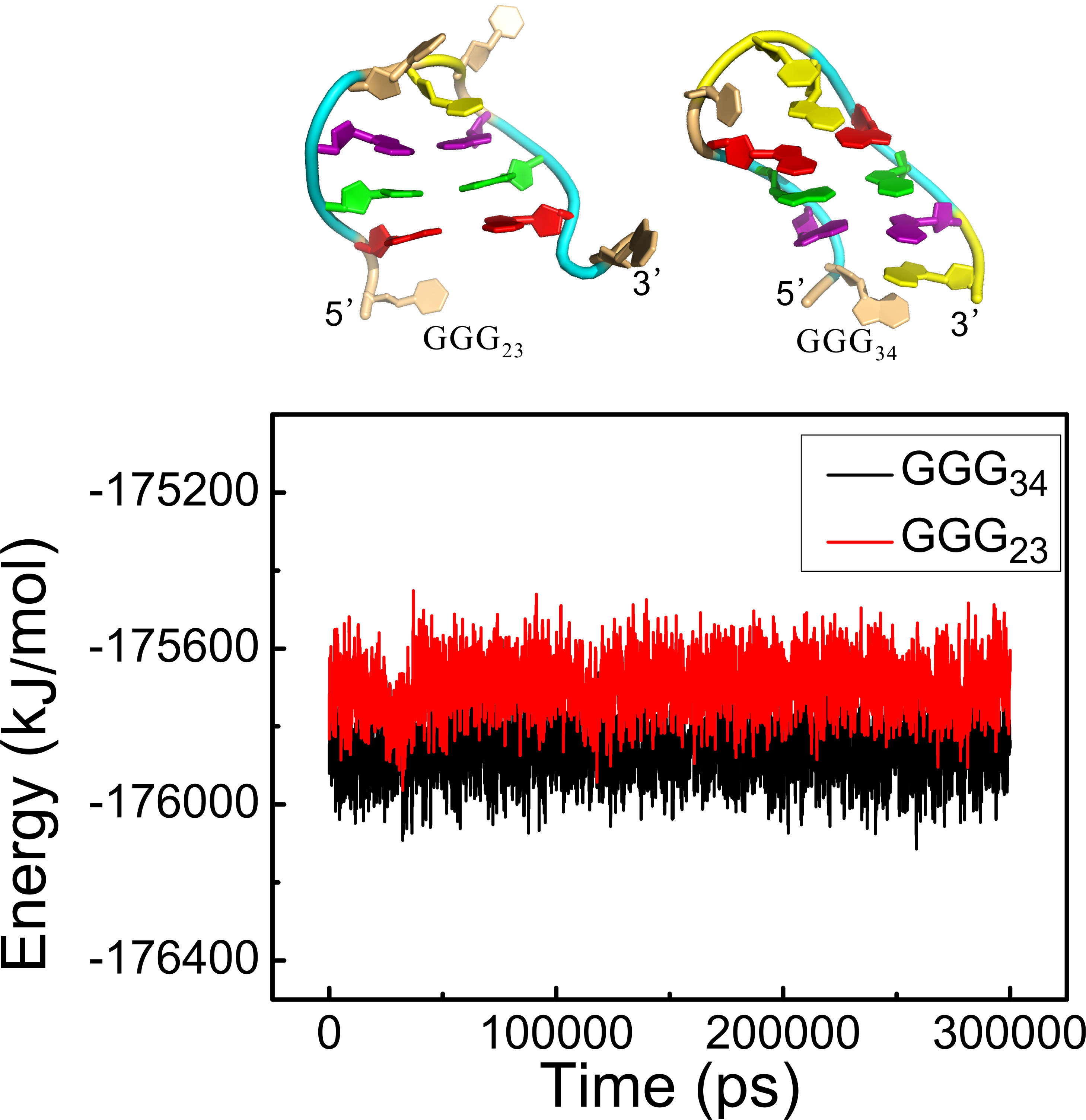

Supplement: Figure S16 — The enthalpy difference between two hairpins and . The two structures shown at the top are the last frames of the simulations. The enthalpy includes both contribution from DNA and water molecules. (TIF) [file pcbi.1003562.s016.tif]
